# Phytochemical Profile and Antioxidant Properties of Invasive Plants *Ailanthus altissima* (Mill.) Swingle and *Helianthus tuberosus* L. in Istria Region, Croatia

**DOI:** 10.3390/antiox14060677

**Published:** 2025-06-03

**Authors:** Mirela Uzelac Božac, Danijela Poljuha, Slavica Dudaš, Josipa Bilić, Ivana Šola, Maja Mikulič-Petkovšek, Barbara Sladonja

**Affiliations:** 1 Department of Agriculture and Nutrition, Institute of Agriculture and Tourism, Karla Huguesa 8, 52440 Poreč, Croatia; mirela@iptpo.hr (M.U.B.); barbara@iptpo.hr (B.S.); 2 Agricultural Department, University of Applied Sciences of Rijeka, Karla Huguesa 6, 52440 Poreč, Croatia; sdudas@veleri.hr; 3 METRIS Research Centre, Istrian University of Applied Sciences, Zagrebačka 30, 52100 Pula, Croatia; jbilic@iv.hr; 4 Department of Biology, Faculty of Science, University of Zagreb, 10000 Zagreb, Croatia; ivana.sola@bio.pmf.hr; 5 Department of Agronomy, Biotechnical Faculty, University of Ljubljana, Jamnikarjeva 101, 1000 Ljubljana, Slovenia; maja.mikulic-petkovsek@bf.uni-lj.si

**Keywords:** extracts, *Jerusalem artichoke*, phenolics, phytochemical profile, tree of heaven

## Abstract

Invasive alien plant species, while ecologically and economically problematic, represent an underutilized source of bioactive phytochemicals with promising phytopharmaceutical applications. This study investigates the LC-DAD-MS phenolic profiles of 70% ethanol and 80% methanol leaf and flower extracts of *Ailanthus altissima* (Mill.) Swingle and *Helianthus tuberosus* L., collected in the Istria region of Croatia, alongside their antioxidant capacities using ABTS, DPPH, and FRAP assays. Both species exhibited high levels of flavonoids and phenolic acids, with consistently higher concentrations in leaf versus flower tissues and in ethanolic versus methanolic extracts. Strong correlations (r > 0.9) between total phenolics and antioxidant activity confirmed the functional significance of these compounds. With a targeted metabolomics approach, in *A. altissima*, 51 phenolics were identified in leaves and 47 in flowers, with ellagitannins predominating; vescalagin isomers reached 94 mg/g DW in leaves and 82 mg/g DW in flowers. *H. tuberosus* extracts contained 34 phenolics in leaves and 33 in flowers, with hydroxycinnamic acids and flavonols dominating; 5-caffeoylquinic acid was the principal compound (25 mg/g DW in leaves, 2 mg/g DW in flowers). The identified phytochemicals are known for their potent antioxidant, anti-inflammatory, anticancer, antimicrobial, and metabolic-regulating properties. Additionally, four leaf-specific compounds were identified in each species, indicating potential for targeted extraction. These findings advance the phytochemical characterization of invasive taxa and highlight their potential as sources of natural antioxidants for functional food and pharmaceutical development.

## 1. Introduction

The continued exploration of plant-derived bioactive compounds plays a vital role in the development of novel phytopharmaceuticals. Phenolics are among the most widely distributed and pharmacologically relevant phytochemicals, noted for their antioxidant, anti-inflammatory, antimicrobial, and anticancer properties. Although ecologically detrimental, invasive alien plant species (IAPSs) are increasingly recognized as rich reservoirs of such bioactive compounds [1,2]. Notably, some studies suggest that IAPSs may produce higher levels of phenolic compounds compared to native species [3] or even their counterparts in native ranges [4]. This phenomenon is hypothesized to result from the novel biochemistry of invasive plants as an adaptive response to new environments and climatic conditions [5].

This study focuses on two IAPSs—*Ailanthus altissima* (Mill.) Swingle and *Helianthus tuberosus* L.—which are widely spread in the Istria region of Croatia. Both species are recognized as invasive alien plant species in Croatia and many other regions due to their aggressive spread and displacement of native flora. They negatively impact biodiversity and ecosystem functioning and can alter soil chemistry [6,7]. Moreover, *A. altissima* produces allelopathic compounds that inhibit the growth of surrounding plants, further exacerbating its invasiveness [8]. Both species have also been associated with adverse effects on human health, including allergenic reactions and potential toxicity [6]. Understanding their phytochemical profiles is thus crucial not only for valorization but also for managing their environmental and health risks.

*A. altissima* (*Simaroubaceae*), widely known as the tree of heaven, is a rapidly proliferating deciduous tree originally native to eastern Asia. First introduced to Europe in the 1700s for ornamental planting [9], it has since established itself on every continent except Antarctica [10]. Due to its aggressive spread and ecological impact, it has been listed as an Invasive Alien Species of Union Concern since 2019 [11]. In Croatia, it forms dense monocultures in urban and disturbed habitats [12]. The species produces allelopathic compounds [13,14,15] and is rich in phenolics, including rutin, caffeic acid, chlorogenic acid, ellagic acid, and resveratrol [16,17,18,19], compounds that are extensively studied for their therapeutic potential. Previous studies have suggested its potential as a source of natural antioxidants and its DNA-protective capacity [16,20,21]. *H. tuberosus*, or *Jerusalem artichoke*, is a perennial plant belonging to the Asteraceae family. It originates from North America and was brought to Europe during the 1600s [22]. It has become invasive in many parts of Central Europe due to its rapid vegetative spread and adaptability to moist, nutrient-rich environments [23]. In Croatia, it is widely distributed along riverbanks and roadsides [12]. The species is known for its edible tubers and its potential in phytotherapeutics and bioethanol production [24]. Its phenolic profile is characterized primarily by chlorogenic acids and related compounds [17,25].

The phenolic composition of plants is influenced by multiple factors, including genotype, phenological stage, plant tissue, extraction method, environmental conditions, and geographic origin [26,27,28]. These variables necessitate site-specific and methodologically tailored analyses to assess phytochemical potential. Previous studies on *A. altissima* leaf extracts obtained through sequential extraction with solvents of varying polarity have demonstrated significant variations in phenolic content and antioxidant activity, influenced by factors such as harvesting season and leaf processing techniques [29]. Similarly, investigations into *H. tuberosus* have shown that phenolic content and antioxidant activity vary across different plant organs, with leaves exhibiting the highest levels [25]. These findings underscore the importance of both solvent selection and plant organ differentiation in phytochemical analyses.

Building on these prior findings and incorporating a regionally focused, organ-specific approach, this study aims to enhance our understanding of the phytochemical potential of these invasive species and their relevance in sustainable resource management. Specifically, we evaluated the phytochemical composition and antioxidant activities of ethanol and methanol extracts derived from the leaves and flowers of *A. altissima* and *H. tuberosus* collected in the Istria region. To our knowledge, this is the first comprehensive analysis of *H. tuberosus* in this geographic area, offering novel insights into its invasive potential and possible applications. In addition, we sought to verify and expand upon our previous preliminary findings for *A. altissima* [16,20].

The study had three main objectives:

(i) To determine total phenolic, flavonoid, and non-flavonoid contents, as well as antioxidant activity, using DPPH, ABTS, and FRAP assays;
(ii) To identify and quantify individual phenolic compounds using LC-DAD-MS;
(iii) To assess the influence of solvent type and plant organ on phytochemical profiles and antioxidant activity through statistical analysis.

## 2. Materials and Methods

### 2.1. Plant Material

Leaves and flowers of *H. tuberosus* and *A. altissima* were collected between June and September of the 2021 growing season in the Istria region of Croatia. A total of 15 samples from each species were obtained from five distinct locations (three samples per site), covering latitudes from N 45.402333 to N 44.846528. Following harvest, the plant material was air-dried at room temperature in the dark. Prior to extraction, individual flowers of *A. altissima* were separated from the inflorescences; therefore, the term “flower extracts” is used throughout this paper to refer specifically to these isolated floral parts.

While our study area is relatively small and geographically homogeneous, future studies should examine site-specific influences to better understand ecological and biochemical variability.

### 2.2. Extraction Procedure

To obtain a representative sample for the study area, 250 g of dry plant material from each collection site was pooled and homogenized using a Grindomix GM 200 knife mill (Retsch, Haan, Germany) set at 10,000 rpm for 30 s.

For spectrophotometric determination of phenolic content, extracts were prepared in triplicate, and analyses were performed following a standardized protocol [30]. In each case, 0.06 g of plant material was dissolved in 2 mL of solvent (70% ethanol or 80% methanol), and then sonicated for 30 min in an ultrasonic bath (40 kHz, 300 W ultrasonic power, 400 W heating power; Holon, Israel). The extracts were subsequently centrifuged at 12,000 rpm for 10 min (Jouan MR23i, Jouan S.A., Saint-Herblain, France), filtered through 0.20 µm PTFE filters (Macherey-Nagel, Düren, Germany), and then stored at +4 °C until analysis.

Phenolic compound extraction for HPLC-DAD-MS analysis followed the protocol of Mikulič Petkovšek et al. [31]. Briefly, 0.2 g of dried plant material was extracted with 6 mL of either 70% ethanol or 80% methanol containing 3% (*v*/*v*) formic acid in a cooled ultrasonic bath for 60 min. Extracts were then centrifuged at 10,000× *g* for 10 min and filtered through 20 µm PTFE filters (Macherey-Nagel, Düren, Germany) prior to analysis.

### 2.3. Total Phenolics, Flavonoids, and Non-Flavonoids Content and Antioxidant Capacity

Total phenolic content (TP) was assessed using the method of Singleton and Rossi [32], while total non-flavonoid content (TNF) was determined following the procedure of Ough and Amerine [33], as detailed by Uzelac et al. [1]. Both methods are based on the reduction of the Folin–Ciocalteu (FC) reagent in the presence of phenolic compounds, resulting in the formation of a molybdenum–tungsten blue complex, which is quantified spectrophotometrically at 765 nm. Results were calculated using a gallic acid calibration curve (y = 0.004x, R^2^ = 0.991 for 70% ethanol extracts; y = 0.009x, R^2^ = 0.996 for 80% methanol extracts, where y is the absorbance at 765 nm and x is the gallic acid concentration in mg/L) and expressed as milligrams of gallic acid equivalents per gram of dry weight (mg GAE/g DW).

Total flavonoid content (TF) was determined according to the method of Martins et al. [34], as adapted by Uzelac et al. [1]. Briefly, 0.02 mL of extract was mixed with 0.88 mL of distilled water, followed by the addition of 0.06 mL of 5% sodium nitrite, 0.06 mL of 10% aluminium chloride, and 0.8 mL of 4% sodium hydroxide. After a 15 min incubation, absorbance was measured at 510 nm. Results were calculated using a catechin calibration curve (y = 0.0024x, R^2^ = 0.993 for 70% ethanol extracts; y = 0.0022x, R^2^ = 0.994 for 80% methanol extracts) and expressed as milligrams of (+)-catechin equivalents per gram of dry weight (mg CE/g DW).

Antioxidant capacity (AC) was evaluated using DPPH, ABTS, and FRAP assays, following the protocols described by Poljuha et al. [35]. DPPH results were calculated using a Trolox standard curve (y = 44.991x, R^2^ = 0.972 for 70% ethanol; y = 47.786x, R^2^ = 0.992 for 80% methanol), while FRAP values were determined from y = 1.638x (R^2^ = 0.993) for ethanol and y = 1.586x (R^2^ = 0.991) for methanol extracts. ABTS values were based on y = 46.137x (R^2^ = 0.981) and y = 41.432x (R^2^ = 0.968), respectively. In all cases, antioxidant activity was expressed as milligrams of Trolox equivalents per gram of dry weight (mg TE/g DW).

All spectrophotometric measurements were conducted in triplicate using a NanoPhotometer P300 (Implen GmbH, München, Germany) with a 2 mL cuvette.

### 2.4. HPLC-DAD-MS Analysis of Phenolic Compounds in Leaf and Flower Extracts

All extracts were analyzed by liquid chromatography with diode-array detection and mass spectrometry (LC-DAD-MS) to identify and quantify individual phenolic compounds, based on reference standards. Separation was performed using a Dionex UltiMate 3000 HPLC system (Thermo Fisher Scientific, San Jose, CA, USA) equipped with a Gemini C18 column (Phenomenex, Torrance, CA, USA) maintained at 25 °C. Detection was carried out using a DAD detector, monitoring at 280 and 350 nm. Phenolic compounds were separated using a binary mobile phase system: mobile phase A consisted of double-distilled water/acetonitrile/formic acid (96.9:3:0.1, *v*/*v*/*v*), and mobile phase B was acetonitrile/double-distilled water/formic acid (96.9:3:0.1, *v*/*v*/*v*). The elution followed a linear gradient: 5–20% B over the first 15 min, and then 20–30% B over 5 min, held isocratically for 5 min, followed by a gradient from 30–90% B over 5 min, and then holding isocratically for 15 min before returning to initial conditions, according to the method described by Mikulič-Petkovšek et al. [36].

A 20 μL injection volume was used, with a flow rate of 0.6 mL/min. Mass spectrometric detection was performed on an LTQ XL Linear Ion Trap MS (Thermo Fisher Scientific, Waltham, MA, USA) equipped with an electrospray ionization (ESI) source in negative mode, using modified parameters based on Mikulič-Petkovšek et al. [37]. Data-dependent full scans were acquired over an *m*/*z* range of 110–1700. Phenolic compounds were identified by comparing fragmentation patterns, retention times, and UV/Vis spectra with those of authentic standards. Quantification was performed based on peak areas, using external standard calibration curves. Results were expressed as milligrams per gram of dry weight (mg/g DW).

Compounds with identical names followed by different numbers (e.g., ‘3-caffeoylquinic acid 1’ and ‘3-caffeoylquinic acid 2’; Table 1) correspond to isomeric forms distinguished by their order of elution on the chromatogram. Since these isomers share the same molecular ion (*m*/*z*) and similar fragmentation patterns, but authentic standards for all forms were not available, we assigned numbers based on retention time sequence. For example, four dicaffeoylquinic acid isomers (*m*/*z* 515) were labeled dicaffeoylquinic acid 1 to 4 according to their elution order and characterized by their MS2 and MS3 fragment ions, consistent with previously reported isomers such as 1,5-diCQA, 3,5-diCQA, and 4,5-diCQA [38]. This approach was similarly applied to other phenolic compounds with multiple isomeric forms, which may represent cis/trans or positional isomers.

### 2.5. Statistical Analysis

**Figure 1:**
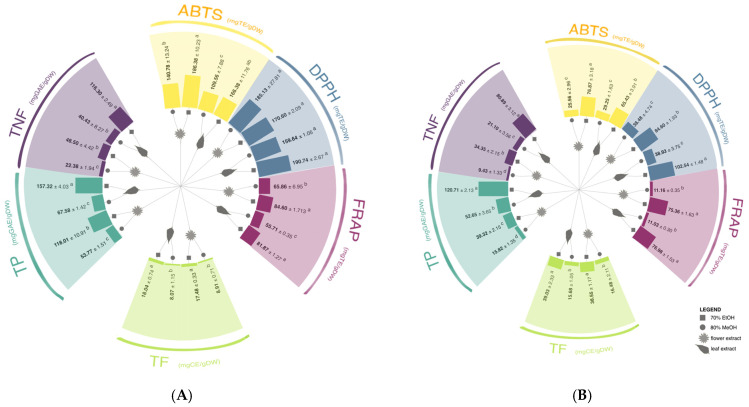
The content of total phenolics (TP), total non-flavonoids (TNF), total flavonoids (TF), and antioxidant capacity (measured by ABTS [2,2′-azino-bis (3-ethylbenzothiazoline-6-sulphonic acid)] radical cation assay, DPPH (2,2-diphenyl-2-picrylhydrazyl) free radical assay, and FRAP (ferric reducing antioxidant power) assay) ABTS, DPPH, and FRAP assays in (**A**) *A. altissima* and (**B**) *H. tuberosus* leaf and flower extracts in two solvents (70% ethanol and 80% methanol). Different letters (a–d) in the same section indicate significant differences between the measured values (two-way ANOVA, Tukey’s test, *p* ≤ 0.01). All results are given in Table S1.

**Figure 2:**
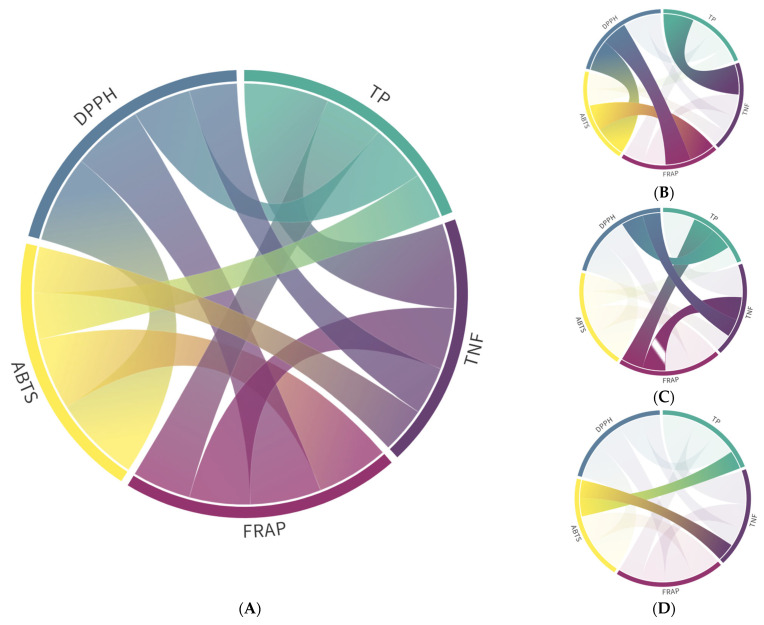
Pearson’s correlation coefficients of total phenolics (TP), non-flavonoids (TNF), and flavonoids (TF) contents and antioxidant capacities determined by ABTS, DPPH, and FRAP assays. (**A**) Only significant correlations (*p* ≤ 0.01; Table S2) are shown. (**B**) Total (0.9 < r < 1.0) and very strong (0.75 < r < 0.9) correlations; (**C**) Strong (0.50 < r < 0.75) correlations; (**D**) Weak correlations (0.25 < r < 0.5).

**Figure 3:**
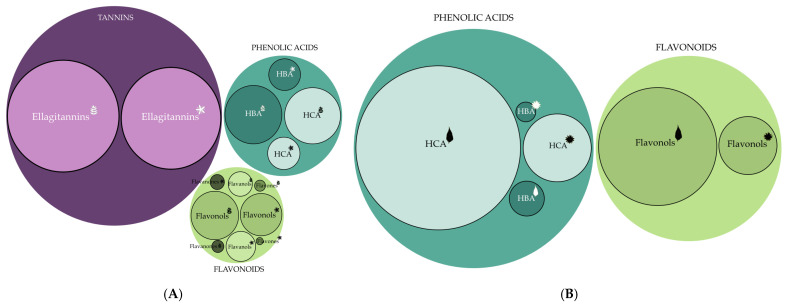
The main phenolic groups obtained by the LC-DAD-MS method in the leaf (and flower ethanolic extracts of (**A**) *Ailanthus altissima* and (**B**) *Helianthus tuberosus*. The ratio of the circles’ sizes corresponds to the total concentration of individual phenolic groups (mg/g of dry weight (DW)). The circle border indicates statistically significant differences between concentrations of individual phenolic groups between the plant organs (one-way ANOVA and LSD test, *p* ≤ 0.01). 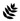 *A. altissima* leaf extract; 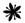 *A. altissima* flower extract; 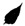 *H. tuberosus* leaf extract; 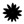 *H. tuberosus* flower extract.

Two-way analysis of variance (ANOVA) with post hoc Tukey’s test was conducted to determine significant influences of factors—organ and solvent) (*p* ≤ 0.05 and 0.01) (Tables S1 and S3). Pearson’s correlation coefficients were calculated to assess the interaction between bio-compounds and antioxidant capacity (Table S2). One-way analysis of variance (ANOVA) with LSD test was conducted to determine significant differences in extracts between the organs in EtOH extracts (Table 1), separately for each species (*p* ≤ 0.05 and 0.01). Data were statistically analyzed using the software Statgraphics Plus 4.0 (Manugistics, Inc., Rockville, MD, USA) and SPSS 23 (IBM, Armonk, NY, USA). Visualization (Figure 1, Figure 2 and Figure 3) was generated using Flourish Studio 1.0.0.3 (Canva, Sidney, Australia) and Sketch version 101 (Eindhoven, The Netherlands).

## 3. Results and Discussion

### 3.1. Phenolic Content and Antioxidant Capacity

The determined contents of total phenolics (TP) and total non-flavonoids (TNF) in all extracts were higher in the leaf than in the flower in both analyzed species (Figure 1, Table S1). However, no statistically significant difference in total flavonoid (TF) content was detected between leaves and flowers.

The highest TP and TNF values were detected in *A. altissima* leaf ethanolic extracts, amounting to 157.3 and 116.3 mg of gallic acid equivalent (GAE)/g of dry weight (DW), respectively (Figure 1, Table S1). In contrast, methanolic leaf extracts of *A. altissima* showed notably lower concentrations (67.6 and 40.4 mg GAE)/g DW, respectively). These findings are lower than values reported in our previous study, where fresh methanolic leaf extracts yielded 247 mg GAE/g DW (TP), 164 mg GAE/g DW (TNF), and 57 mg catechin equivalents (CE)/g DW (TF) [16]. Comparable results were observed by Mohamed et al. [19], who reported a TP value of 209.8 mg GAE/g DW in 85% methanol leaf extracts of *A. altissima*. Variations in these values can be attributed to differences in the year of plant collection, climatic conditions, plant maturity, sampling location, and extraction methods. Supporting this, Luís et al. [39] measured 136.6 mg GAE/g DW in ethanolic leaf extracts of *A. altissima*. Further highlighting this variability, Cocîrlea et al. [29] demonstrated that harvest season and processing method significantly impact polyphenol content. They found the highest TP in frozen autumn-harvested leaves (72.5 mg GAE/g DW) and the highest TF in summer-harvested leaves (91.9 mg QE/g DW). In the present study, TF content ranged from 8.1 to 8.9 mg CE/g DW in ethanolic extracts, and from 17.5 to 18 mg CE/g DW in methanolic extracts—values significantly lower than the 87.1 mg CE/g DW reported by Luís et al. [39].

For *H. tuberosus*, the highest TP was found in ethanolic leaf extracts (120.7 mg GAE/g DW), followed by 52.6 mg GAE/g DW in methanolic leaf extracts. Flower extracts yielded lower TP values regardless of the solvent used (39.3 mg GAE/g DW in ethanol; 15.8 mg GAE/g DW in methanol) (Figure 1B, Table S1). These findings align with the study of Sreekanth and Devi [40], who reported 87–127 mg GAE/g DW in ethanolic leaf extracts. Alyas et al. [41] reported a total phenolic content of 30.6 mg GAE/g in tuber extracts prepared with 70% ethanol, which is lower than the values observed in our research. This difference underscores the impact that both the choice of plant organ and extraction technique can have on phenolic yield. Their findings further indicate that autoclave-assisted extraction may enhance the recovery of antioxidants from *Jerusalem artichoke* compared to conventional aqueous or 70% ethanol extraction methods. Furthermore, variations in observed values, as in the case of *A. altissima*, could be attributed to the plant maturity stage. Chen et al. [42] reported the variation in the total phenolic content of *H. tuberosus* ethanol leaf extracts depending on the growth stage, which reached its highest concentration of 5.3 mg/g DW at the flowering stage. The choice of extraction method, especially the solvent polarity, also significantly influences the total phenolic content and antioxidant capacity (AC). Ethanol and methanol, as polar solvents, are more effective in extracting phenolic compounds than less polar alternatives. Do et al. [43] and Showkat et al. [25] emphasized that solvent polarity strongly influences phenolic extraction efficiency, particularly for compounds such as caffeoylquinic acid isomers. Moreover, methanol is known to preferentially extract lower-molecular-weight phenolics, while ethanol, being food-safe and broadly effective, presents a practical choice for extraction purposes [43]. Yuan et al. [44] found that an ethyl acetate *H. tuberosus* leaf fraction, with intermediate polarity, yielded 266.7 mg GAE/g DW, further underscoring the relevance of solvent characteristics. Another common practice in scientific papers is to present results in different formats, which can hinder direct comparison with existing literature.

Regarding antioxidant activity, the highest AC values for *A. altissima* were observed in ethanolic leaf extracts using the DPPH assay (190.7 mg TE/g DW) and in ethanolic flower extracts using ABTS (186.4 mg TE/g DW) (Figure 1, Table S1). Mohamed et al. [19] reported a DPPH SC_50_ of 22.00 µg/mL for 85% methanol extracts, while Andonova et al. [21] noted DPPH and ABTS activities of 225.6 and 299.5 mmol TE/g DW, respectively, in ethanolic aerial part extracts. For *H. tuberosus*, the highest AC values were again found in ethanolic extracts: 102.5 mg TE/g DW (leaf) and 84.6 mg TE/g DW (flower), both measured using the DPPH assay (Figure 1, Table S1).

Generally, ethanolic extracts outperformed methanolic ones in terms of AC, and leaf extracts often showed higher AC than flower extracts, as later confirmed by PCA analysis, which revealed clear separation between extraction solvents and plant organs. (Ethanol is a Generally Recognized As Safe (GRAS) solvent widely accepted for food and pharmaceutical applications due to its regulatory approval and efficacy in extracting bioactive phenolics [45]. In the US, ethanol is approved for specific food uses such as antimicrobial and preservative applications, while in the EU it is not listed as a food additive under Regulation (EC) No. 1333/2008 but is commonly used as a solvent in flavoring preparations. However, products using ethanol extraction may require novel food authorization if introduced to the EU market post-1997. Given its safety profile and extraction efficiency, ethanol is preferable over methanol for developing functional food and phytopharmaceutical formulations.

Strong and significant correlations were observed between all three antioxidant assays, as well as between TP and TNF (r = 0.9–1.0, Table S2, Figure 2A,B). Additionally, TP and TNF were strongly correlated with AC as determined by FRAP and DPPH (0.5 < r < 0.75), with slightly lower correlations for ABTS (r ≈ 0.49) (Table S2, Figure 2A,D).

To evaluate antioxidant efficiency, we normalized total antioxidant capacity to total phenolic content (mg TE/mg GAE). TE/GAE ratios varied across extracts and assays (Table S1). The highest ABTS efficiency was in *H. tuberosus* leaf extract (70% EtOH, 1.95), while *A. altissima* flower extract (80% MeOH) also showed a high value (2.62). DPPH/TP ratios exceeded 2.3 for both *A. altissima* flower (80% MeOH) and *H. tuberosus* leaf (70% EtOH). FRAP/TP values were generally lower, except in *H. tuberosus* leaf extract (70% EtOH, 1.92), indicating strong reducing power per phenolic unit. Similar conclusions can be noted regarding the separation of solvents in PCA analysis and correlation between specific compounds and antioxidant methods (Tables S2–S4). These differences highlight the influence of plant species and extraction solvent. Variations among ABTS, DPPH, and FRAP also reflect assay principles: ABTS and DPPH measure radical scavenging (electron/hydrogen transfer), while FRAP assesses reducing power via iron reduction. For example, *A. altissima* flower extract showed high ABTS/TP and DPPH/TP but moderate FRAP/TP, suggesting strong scavenging but lower reducing ability. In contrast, *H. tuberosus* leaf extract was consistently efficient across all methods, indicating a broader antioxidant profile. Overall, both phenolic content and functional activity depend on the compound type, extraction method, and assay used. While colorimetric assays provide initial antioxidant screening, future studies should integrate cell-based, ex vivo, or in vivo models to evaluate bioactivity in physiologically relevant systems. *A. altissima* and *H. tuberosus* leaves, with their high phenolic yields and antioxidant efficiency, are prioritized for downstream applications such as nutraceutical formulations or stability testing during in vitro digestion. Our preliminary results on the anti-inflammatory, genotoxic, and cytotoxic effects of the extracts on various cell lines, as well as their antimicrobial activity, indicate the potential for further development of phytopharmaceutical applications. Furthermore, our initial findings [46] demonstrated that *A. altissima* plant extracts modulate GST activity in HEPG2 cell lysates and extracellular media, underscoring the therapeutic potential of this and other invasive species and their specialized metabolites. These results warrant further investigation into their regulatory effects on GST activity and their prospective role in cancer therapy strategies.

### 3.2. Identification and Quantification of Phenolic Compounds

Based on external standards, a total of 67 phenolic compounds were identified in the analyzed extracts: 51 in the leaf and 47 in the flower of *A. altissima*, and 34 in the leaf and 33 in the flower of *H. tuberosus* (Table 1). The detected compounds encompassed phenolic acids, specifically derivatives of hydroxycinnamic acid (HCA) and hydroxybenzoic acid (HBA), along with various flavonoids—including flavones, flavonols, flavanols, and flavanones—as well as non-flavonoid constituents like ellagitannins. The influence of the plant organ exceeded that of the solvent across a majority of phenolic groups, with the exception of ellagitannins in *A. altissima*, where the solvent had a more pronounced impact, and flavanols, flavonols, and flavanones, where neither organ nor solvent had an impact (Table S5). Therefore, to streamline interpretation, Table 1 presents only the results from 70% ethanol extracts, while the full data (including 80% methanol) are available in Supplementary Table S5.

In *A. altissima*, leaf extracts showed a significantly higher total phenolic content than flower extracts (189.5 vs. 129.1 mg/g DW; Table 1, Figure 3A), with the same trend observed in methanolic extracts, although in higher concentrations (234.8 mg/g DW in leaf and 163.1 mg/g DW in flower; Table S5). In *H. tuberosus*, the total detected phenolics concentrations in ethanolic extracts were lower (65.8 for leaf and 12.1 mg/g of DW for flower; Table 1, Figure 3B), and there were no statistically significant differences between values observed in methanolic extracts (Table S5). These findings confirm the role of the plant organ as a critical determinant of phytochemical richness.

In total, 29 species-specific compounds for *A. altissima* and 16 for *H. tuberosus* were identified (Table 1). Four compounds were organ-specific for *A. altissima* leaf (3-caffeoylquinic acid 2, 5-caffeoylquinic acid 2, procyanidin dimer 2, and quercetin-3-rutinoside). In *H. tuberosus,* seven compounds were organ-specific—four for leaf (4-*p*-coumaroylquinic acid 2, 5-*p*-coumaroylquinic acid 1, gallic acid, and quercetin-rhamnosylhexoside) and three for flower (quercetin pentoside 1, quercetin pentoside 2, and quercetin malosyl hexoside) (Table 1).

**Table 1:** Phenolic compounds (mg/g of dry weight (DW)) of *A. altissima* and *H. tuberosus* leaf and flower extracts identified by LC-DAD-MS in 70% ethanol. Values represent the mean ± SD of four replicates. Different letters (a, b, A, B) in the same row indicate significant intra-species differences, determined by one way-ANOVA and LSD test; *p*-value ≤ 0.01; n.d.—not detected.

Phenolic Compounds	*A. altissima*		*H. tuberosus*	
	Leaf	Flower	Leaf	Flower
3-caffeoylquinic acid 1	0.975 ± 0.275 ^a^	0.924 ± 0.190 ^a^	0.026 ± 0.004 ^A^	0.405 ± 0.009 ^B^
3-caffeoylquinic acid 2	2.917 ± 0.122	n.d.	n.d.	n.d.
4-caffeoylquinic acid 1	0.543 ± 0.119 ^a^	0.100 ± 0.017 ^b^	1.336 ± 0.183 ^A^	0.190 ± 0.064 ^B^
4-caffeoylquinic acid 2	3.112 ± 0.530 ^a^	0.449 ± 0.015 ^b^	n.d.	n.d.
5-caffeoylquinic acid 1	5.298 ± 0.474 ^a^	1.721 ± 0.421 ^b^	25.341 ± 1.831 ^A^	2.052 ± 0.261 ^B^
5-caffeoylquinic acid 2	9.080 ± 1.203	n.d.	0.673 ± 0.061 ^A^	0.097 ± 0.011 ^B^
Caffeic acid	1.413 ± 0.314 ^a^	0.426 ± 0.029 ^b^	0.086 ± 0.014 ^A^	0.100 ± 0.012 ^A^
Caffeic acid hexoside 1	0.374 ± 0.057 ^a^	0.089 ± 0.015 ^b^	0.439 ± 0.052 ^A^	0.565 ± 0.109 ^A^
Caffeic acid hexoside 2	0.273 ± 0.086 ^b^	1.563 ± 0.100 ^a^	0.164 ± 0.024 ^A^	0.148 ± 0.024 ^A^
Dicaffeoylquinic acid 1	n.d.	n.d.	0.551 ± 0.111 ^A^	0.205 ± 0.008 ^B^
Dicaffeoylquinic acid 2	n.d.	n.d.	5.880 ± 0.584 ^A^	1.079 ± 0.043 ^B^
Dicaffeoylquinic acid 3	n.d.	n.d.	0.599 ± 0.091 ^A^	0.475 ± 0.019 ^B^
Dicaffeoylquinic acid 4	n.d.	n.d.	0.703 ± 0.047 ^A^	0.438 ± 0.077 ^B^
*p*-coumaric acid hexoside 1	0.461 ± 0.098 ^a^	0.192 ± 0.033 ^b^	n.d.	n.d.
3 *p*-coumaroylquinic acid	0.346 ± 0.090 ^a^	0.005 ± 0.001 ^b^	3.623 ± 0.485 ^A^	0.240 ± 0.013 ^B^
4-*p*-coumaroylquinic acid 1	0.788 ± 0.053 ^a^	0.443 ± 0.038 ^b^	0.153 ± 0.025 ^A^	0.078 ± 0.006 ^B^
4-*p*-coumaroylquinic acid 2	n.d.	n.d.	0.095 ± 0.014	n.d.
5-*p*-coumaroylquinic acid 1	0.530 ± 0.080 ^a^	0.424 ± 0.016 ^b^	0.485 ± 0.050	n.d.
5-*p*-coumaroylquinic acid 2	n.d.	n.d.	0.315 ± 0.043 ^A^	0.111 ± 0.025 ^B^
3-feruloylquinic acid	0.262 ± 0.067 ^a^	0.399 ± 0.013 ^a^	0.055 ± 0.008 ^A^	0.115 ± 0.035 ^B^
4-feruloylquinic acid	0.028 ± 0.002 ^b^	0.523 ± 0.026 ^a^	n.d.	n.d.
5-feruloylquinic acid 1	0.021 ± 0.003 ^b^	0.830 ± 0.032 ^a^	0.431 ± 0.036 ^A^	0.242 ± 0.025 ^B^
5-feruloylquinic acid 2	n.d.	n.d.	0.189 ± 0.024 ^A^	0.134 ± 0.038 ^B^
**Hydroxycinnamic acid** **derivatives**	**26.771 ± 1.684 ^a^**	**8.086 ± 0.444 ^b^**	**41.126 ± 2.651 ^A^**	**6.615 ± 0.674 ^B^**
Gallic acid	0.531 ± 0.081 ^b^	0.947 ± 0.059 ^a^	0.080 ± 0.193	n.d.
Protocatechuic acid	0.026 ± 0.003 ^b^	0.861 ± 0.106 ^a^	1.503 ± 0.308 ^A^	0.404 ± 0.060 ^B^
Ellagic acid	22.486 ± 2.276 ^A^	2.125 ± 0.397 ^b^	n.d.	n.d.
Ellagic acid pentoside 1	4.948 ± 0.494 ^A^	3.074 ± 0.641 ^b^	n.d.	n.d.
Ellagic acid pentoside 2	0.042 ± 0.008 ^b^	0.689 ± 0.063 ^a^	n.d.	n.d.
**Hydroxybenzoic acid** **derivatives**	**28.037 ± 2.767 ^a^**	**7.693 ± 0.881 ^b^**	**1.583 ± 0.292 ^A^**	**0.404 ± 0.060 ^B^**
Procyanidin dimer 1	0.029 ± 0.007 ^b^	0.204 ± 0.050 ^a^	n.d.	n.d.
Procyanidin dimer 2	4.373 ± 0.529	n.d.	n.d.	n.d.
Epicatechin	0.020 ± 0.003 ^b^	5.661 ± 0.280 ^a^	n.d.	n.d.
Gallocatechin	2.331 ± 0.203 ^a^	0.790 ± 0.131 ^b^	n.d.	n.d.
**Flavanols**	**6.799 ± 0.526 ^a^**	**6.838 ± 0.272 ^a^**	**n.d.**	**n.d.**
Quercetin pentoside 1	0.336 ± 0.035 ^a^	0.286 ± 0.040 ^b^	n.d.	0.132 ± 0.027
Quercetin pentoside 2	0.011 ± 0.002 ^b^	0.032 ± 0.007 ^a^	n.d.	0.138 ± 0.011
Quercetin-3-rutinoside	0.001 ± 0.000	n.d.	1.279 ± 0.196 ^A^	0.262 ± 0.075 ^B^
Quercetin-3-galactoside	1.926 ± 0.195 ^b^	2.634 ± 0.366 ^a^	0.489 ± 0.085 ^A^	0.124 ± 0.019 ^B^
Quercetin-3-glucoside	8.031 ± 0.813 ^a^	2.940 ± 0.550 ^b^	0.760 ± 0.073 ^A^	0.422 ± 0.050 ^B^
Quercetin-3-glucuronide	n.d.	n.d.	5.011 ± 0.227 ^A^	1.634 ± 0.140 ^B^
Quercetin-3-rhamnoside	0.140 ± 0.026 ^b^	1.004 ± 0.075 ^a^	n.d.	n.d.
Quercetin galloylhexoside 1	0.283 ± 0.032 ^b^	0.617 ± 0.053 ^a^	n.d.	n.d.
Quercetin galloylhexoside 2	0.929 ± 0.179 ^a^	0.953 ± 0.087 ^a^	n.d.	n.d.
Quercetin acetylhexoside 1	3.469 ± 0.346 ^a^	1.010 ± 0.109 ^b^	n.d.	n.d.
Quercetin acetylhexoside 2	0.175 ± 0.023 ^b^	0.363 ± 0.030 ^a^	n.d.	n.d.
Quercetin-rhamnosylhexoside	n.d.	n.d.	0.235 ± 0.006	n.d.
Quercetin malosyl hexoside	n.d.	n.d.	n.d.	0.558 ± 0.004
Isorhamnetin hexoside	n.d.	n.d.	0.240 ± 0.016 ^A^	0.200 ± 0.032 ^A^
Isoramnetin hexosylpentoside	n.d.	n.d.	0.470 ± 0.035 ^A^	0.351 ± 0.063 ^B^
Isorhamnetin glucuronide	n.d.	n.d.	0.374 ± 0.025 ^A^	0.184 ± 0.043 ^B^
Isorhamnetin acetylhexoside	0.214 ± 0.059 ^b^	0.555 ± 0.113 ^a^	n.d.	n.d.
Kaempferol hexoside 1	1.190 ± 0.120 ^b^	2.075 ± 0.288 ^a^	0.141 ± 0.007 ^A^	0.117 ± 0.026 ^A^
Kaempferol hexoside 2	0.751 ± 0.119 ^a^	0.362 ± 0.074 ^b^	5.417 ± 0.232 ^A^	0.055 ± 0.002 ^B^
Kaempferol rhamnosylhexoside 1	0.087 ± 0.017 ^b^	0.282 ± 0.034 ^a^	0.048 ± 0.005 ^A^	0.036 ± 0.0047 ^B^
Kaempferol rhamnosylhexoside 2	0.123 ± 0.021 ^b^	0.304 ± 0.069 ^a^	n.d.	n.d.
Kaempferol-3-rutinoside	n.d.	n.d.	0.918 ± 0.155 ^A^	0.211 ± 0.072 ^B^
Kaempferol-3-glucuronide	n.d.	n.d.	7.125 ± 0.639 ^A^	0.420 ± 0.054 ^B^
Kaempferol acetylhexoside 1	n.d.	n.d.	0.474 ± 0.078 ^A^	0.132 ± 0.026 ^B^
Kaempferol acetylhexoside 2	0.736 ± 0.072 ^a^	0.594 ± 0.073 ^a^	n.d.	n.d.
Kaempferol galloylhexoside	0.202 ± 0.035 ^a^	0.134 ± 0.029 ^b^	n.d.	n.d.
**Flavonols**	**18.521 ± 1.489 ^a^**	**14.119 ± 1.376 ^b^**	**23.132 ± 1.197 ^A^**	**4.973 ± 0.244 ^B^**
Naringenin hexoside 1	0.366 ± 0.048 ^b^	0.656 ± 0.077 ^a^	n.d.	n.d.
Naringenin hexoside 2	0.033 ± 0.007 ^a^	0.019 ± 0.002 ^b^	n.d.	n.d.
Naringenin hexoside 3	0.046 ± 0.008 ^a^	0.023 ± 0.002 ^b^	n.d.	n.d.
Naringenin hexoside 4	0.332 ± 0.053 ^b^	0.572 ± 0.033 ^a^	n.d.	n.d.
**Flavanones**	**0.776 ± 0.086 ^b^**	**1.269 ± 0.086 ^a^**	**n.d.**	**n.d.**
Vescalagin isomer 1	80.705 ± 4.531 ^a^	54.298 ± 6.480 ^b^	n.d.	n.d.
Vescalagin isomer 2	13.442 ± 0.706 ^b^	27.364 ± 1.128 ^a^	n.d.	n.d.
HHDP galloylhexose	11.989 ± 1.830 ^a^	8.659 ± 0.500 ^b^	n.d.	n.d.
HHDP digalloylhexose isomer	1.749 ± 0.3436 ^a^	0.715 ± 0.029 ^b^	n.d.	n.d.
**Ellagitannins**	**107.431 ± 4.019 ^a^**	**91.035 ± 6.712 ^b^**	**n.d.**	**n.d.**
Apigenin hexoside	0.669 **±** 0.107 ^a^	0.142 **±** 0.010 ^b^	n.d.	n.d.
**Flavones**	**0.669 ± 0.107 ^a^**	**0.142 ± 0.010 ^b^**	**n.d.**	**n.d.**
**TOTAL**	**189.541 ± 9.473 ^a^**	**129.182 ± 7.002 ^b^**	**65.841 ± 0.560 ^A^**	**12.048 ± 0.741 ^B^**

The external standards used: caffeic acid, apigenin-7-glucoside, ferulic acid, quercetin-3-*O*-rhamnoside, neochlorogenic (3-caffeoylquinic) acid, naringenin, ellagic acid, gallic acid, chlorogenic acid, and rutin (quercetin-3-*O*-rutinoside); (-)epicatechin, quercetin-3-*O*-galactoside, quercetin-3-*O*-glucoside, *p*-coumaric acid, procyanidin B1, and kaempferol-*O*-glucoside; quercetin-3-*O*-xyloside and quercetin-3-*O*-arabinopyranoside; and isorhamnetin-3-*O*-glucoside.

The highest numbers of individual compounds for leaf and flower extracts in *H. tuberosus* were identified in the HCA group (19 and 16, respectively), followed by flavonols (14 and 16, respectively) (Table 1). Kaszás et al. [47] also identified 18 flavonoids in leaf extracts of the same species, noting that the samples were mechanically pressed into a green juice. This process retained the vacuoles, which are the primary storage sites for soluble flavonoids. A similar distribution of the individual compounds was found for *A. altissima* leaf and flower extracts, with a dominant HCA group (16 and 14, respectively) and flavonols (17 and 16, respectively) (Table 1). Compared to our previous research [20], this study identified a higher number of individual phenolic compounds within the main groups and higher overall concentrations in those groups. The observed difference could be attributed to variations in sampling locations and climate conditions. In other research on aerial parts of *A. altissima* in Portugal [39], HCA were the dominant group of phenolics.

**Figure 4:**
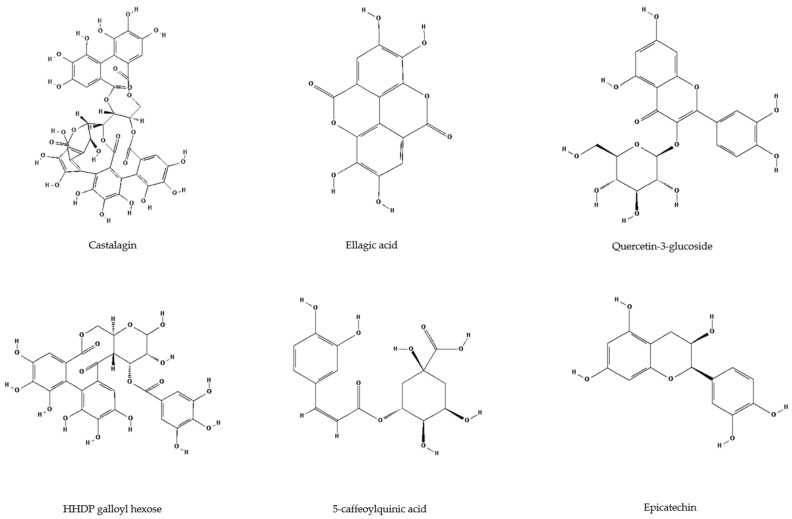
The structures of the main compounds found in leaf and flower extracts of *A. altissima* (PubChem; available online: https://pubchem.ncbi.nlm.nih.gov/ (accessed on 20 May 2025)).

Ellagitannins dominated *A. altissima* extracts (56–70% of total phenolics; Table 1, Figure 3A), with vescalagin (castalagin) isomers emerging as the major compounds in both leaves and flowers (Table 1, Figure 4). Vescalagin isomers in leaves (94 mg/g DW) and flowers (82 mg/g DW) exceeded the concentrations reported in prior studies [20]. This compound has previously been associated with oak and chestnut species [48]. It is known to have the potential to prevent the progression of diabetes mellitus, antitumor properties, and bactericidal activity against methicillin-resistant bacteria, including MRSA and MRSE [49,50,51]. Ellagic acid derivatives and HHDP galloyl hexose represented some of the predominant constituents in the leaf extracts, with concentrations of 22.5 mg/g DW and 11.9 mg/g DW, respectively. Notably, HHDP galloyl hexose was also found in significant amounts in the flower extracts, reaching 8.7 mg/g DW (Table 1; Figure 4). These naturally occurring phenolics are common in various plant species, particularly fruits like cranberries [52]. Ellagic acid (Figure 4), isolated from an 85% methanol extract of *A. altissima* leaves, demonstrated strong antiradical activity (IC_50_ = 2.26 µg/mL), outperforming ascorbic acid (IC_50_ = 6.44 µg/mL) [19]. The flavonol quercetin-3-glucoside was detected in leaf ethanol extracts at 8.03 mg/g DW. Known for its protective effects against alcohol-induced liver damage, this compound has been studied for its hepatoprotective properties [53]. In *A. altissima* flower extracts, epicatechin was notably abundant (5.6 mg/g DW) (Table 1). Previous studies reported lower concentrations in leaves and flowers (0.25 and 0.14 mg/g DW, respectively) [17], and similar findings were confirmed by Andonova et al. [21], who recorded the highest levels in stems (0.54 mg/g DW). Epicatechin (Figure 4), a flavanol found in tea, cocoa, and various fruits and vegetables, has been linked to cardiovascular, metabolic, and neuroprotective benefits [54,55]. Beyond human health, it may also function as an allelochemical, affecting soil biota and chemistry [17].

**Figure 5:**
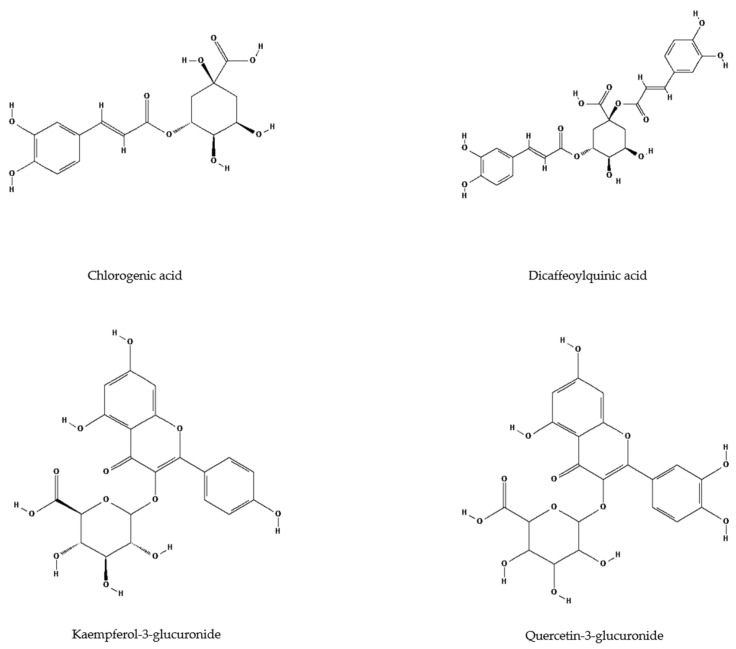
The structures of four main compounds found in leaf and flower extracts of *H. tuberosus* (PubChem; available online: https://pubchem.ncbi.nlm.nih.gov/ (accessed on 20 May 2025)).

Chlorogenic acid isomers, a key subgroup of non-flavonoid phenolics, are characteristic of the Asteraceae family and were abundant in *H. tuberosus* extracts [47]. Hydroxycinnamic acids dominated the leaf and flower extracts, accounting for 62% and 55% of total phenolics, respectively (Table 1, Figure 3B). The primary compound in both was 5-caffeoylquinic acid 1 (chlorogenic acid; Figure 5), present at 25.3 mg/g DW in leaves and 2.05 mg/g DW in flowers (Table 1). Previous studies also identified chlorogenic acid as the main compound in leaf and tuber ethanol extracts [25,56,57,58]. Due to its chemical instability, chlorogenic acid readily forms isomers, including other caffeoylquinic acids, dicaffeoylquinic acids, and hydroxycinnamic acid–quinic acid complexes [59,60]. These isomers maintain strong biological activity, particularly anti-inflammatory and antioxidant effects [61]. Zhang and Kim [62] confirmed the antioxidant potential of *H. tuberosus* tuber extract on A549 human lung epithelial cells. Similarly, Jantaharn et al. [63] reported moderate growth inhibition of colon cancer HCT116 cells by phenolics such as ent-kaurenoic acid, 8-methoxyobiquin, and isoliquiritigenin from flower extracts. According to Zhu et al. [64], treatment with leaf extracts led to a reduction in oxidative stress within cultured human neuroblastoma HTB-11 cells. Besides chlorogenic acid, other caffeic acid derivatives (Figure 5) also contributed to antioxidant activity. Dicaffeoylquinic acid 2 was found in leaf extracts at 5.88 mg/g DW (Table 1, Figure 5). Compounds such as 1,5- and 3,5-dicaffeoylquinic acids demonstrated strong radical-scavenging activity in DPPH and ABTS assays, with SC_50_ values between 2.79 and 5.08 µM [44]. Among flavonoids, kaempferol-3-glucuronide (7.13 mg/g DW) and quercetin-3-glucuronide (5.01 mg/g DW) were the major compounds in leaf extracts (Table 1, Figure 5).

Glucose and glucuronic acid are among the most common sugar substituents in these compounds [65]. Flavonoid glucuronides like quercetin-3-O-glucuronide are noted for their anti-inflammatory and neuroprotective properties [66]. Flavonoids also act as antioxidants by donating electrons or protons to neutralize reactive oxygen species and halt lipid peroxidation, or by chelating pro-oxidant metals [67,68].

**Figure 6:**
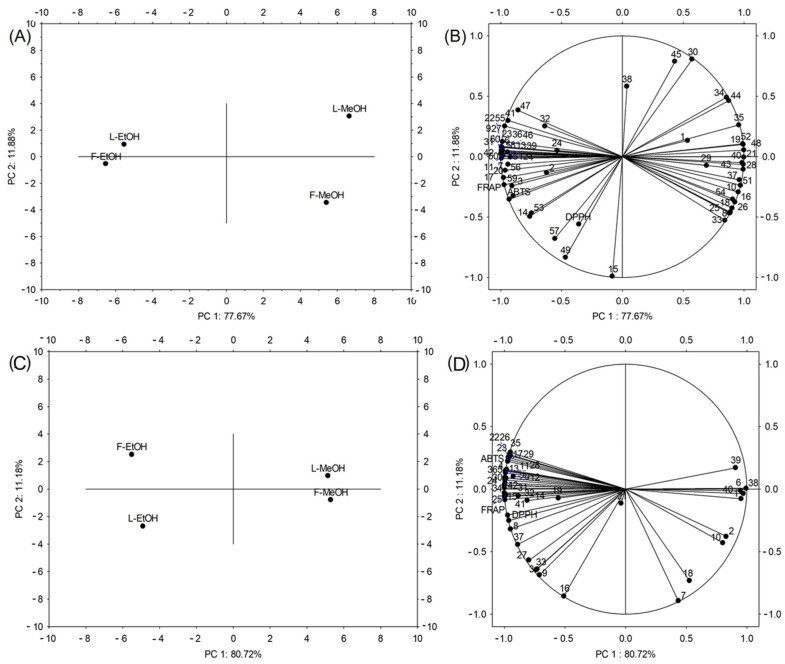
Principal component analysis (PCA) of the individual metabolites in leaf (L) and flower (F) extracts in two solvents (EtOH, MeOH) and antioxidant capacity measured by three assays (ABTS, DPPH, FRAP) in (**A**,**B**) *Ailanthus altissima* and (**C**,**D**) *Helianthus tuberosus*. Numbers (1–60) are related to the individual identified phenolics, as depicted in the Table S6.

PCA was performed to identify the phenolic compounds most responsible for antioxidant capacity (Figure 4). For *A. altissima*, the first two principal components explained 89.55% of the total variance (PC1: 77.67%, PC2: 11.88%), while for *H. tuberosus*, they accounted for 91.9% (PC1: 80.72%, PC2: 11.18%). Clear separation was observed between extraction solvents along PC1, with ethanol extracts showing higher antioxidant capacity than methanol extracts for both species. Additionally, organ-specific separation was evident along PC2, distinguishing flower from leaf extracts. The loadings plots (Figure 6B,D) revealed that ABTS, DPPH, and FRAP assays clustered together in the positive PC1 direction, indicating their strong intercorrelation. For *A. altissima*, compounds 2, 3, 7, 11, 17, 20, 56, and 59 loaded in the same direction as the antioxidant assays, suggesting these phenolics (primarily ellagitannins and HCA derivatives) are the major contributors to antioxidant capacity, confirming the correlations presented in Tables S2 and S3. In *H. tuberosus*, compounds 8, 11, 14, 15, 17, 19, 25, 28, and 29 (predominantly hydroxycinnamic acids and flavonols, including chlorogenic acid derivatives) showed the strongest associations with antioxidant activity. These results confirm that ellagitannins in *A. altissima* and hydroxycinnamic acids in *H. tuberosus* are the primary bioactive compounds responsible for the observed antioxidant properties.

*A. altissima*, traditionally a valuable medicinal resource in Asia, possesses the potential to treat a wide variety of ailments. Despite its widespread presence in Croatia and Europe in general, it holds limited economic importance. Its economic use remains limited, primarily to honey production and soil remediation [8]. Nevertheless, its demonstrated antimicrobial and antioxidant activities suggest promising pharmaceutical applications [16]. Conversely, *H. tuberosus* has a longer tradition of local use, with its tubers consumed as food and recognized for their functional properties [69]. Comparative analysis with published data from other Balkan regions, including Serbia, Romania, and Bulgaria [17,21,70], reveals that our Istrian samples of *A. altissima* and *H. tuberosus* generally contain higher concentrations of some key phenolic compounds, such as caffeic acid, rutin, epicatechin, and quercetin and kaempferol derivatives. These findings suggest that specific environmental factors in the Istrian region may promote the accumulation of bioactive phenolics, contributing to the distinct phytochemical profiles observed in these invasive species. Overall, our findings underscore the potential to valorize *A. altissima* and *H. tuberosus* as sources of bioactive compounds and functional foods, supporting local economies and advancing sustainable management strategies.

In addition to their potential value as sources of bioactive compounds and functional foods, the targeted and sustainable utilization of these species’ biomass could serve as a complementary approach to controlling their spread. Harvesting these species for phytopharmaceutical or food applications does not promote their cultivation but rather supports their removal from natural habitats, aligning with integrated invasive species management strategies that emphasize both ecological restoration and resource recovery. This dual approach can help mitigate the negative impacts of invasive plants on biodiversity and ecosystem function, while also providing economic and societal benefits.

## 4. Conclusions

This study presents the first comprehensive phytochemical profiling of *Ailanthus altissima* flowers and both leaf and flower extracts of *Helianthus tuberosus* from the Istrian region, Croatia, expanding on our previous work on *A. altissima* leaves. Through targeted LC-DAD-MS analysis and multiple antioxidant assays, we confirmed high concentrations of phenolic compounds—particularly ellagitannins in *A. altissima* and hydroxycinnamic acids in *H. tuberosus*—that corresponded strongly with antioxidant capacity. The identification of 45 species-specific and 8 organ-specific phenolic compounds underscores the phytochemical uniqueness of both taxa. Aside from the leaves, vescalagin isomers were dominant in *A. altissima* flowers also, marking the first report of their occurrence in the flowers of this species in Croatia, while chlorogenic acid was confirmed as the primary antioxidant compound in *H. tuberosus*. Strong correlations (r > 0.9) were observed between phenolic content and antioxidant activity, particularly in ethanolic extracts and leaf tissues, suggesting these combinations are most promising for future applications. These findings align with all three objectives, providing robust evidence that invasive alien plant species represent an underutilized but valuable resource for sustainable phytopharmaceutical development. Local communities could leverage these invasive species for antioxidant-rich extracts, aligning with circular economy principles by transforming ecological threats into commercial resources. This research not only advances the phytochemical understanding of two ecologically significant species but also highlights their practical relevance in natural antioxidant discovery, potentially guiding future functional food, cosmetic, and pharmaceutical innovations.

## Data Availability

The original contributions presented in this study are included in the article. Further inquiries can be directed to the corresponding author(s).

## Supplementary Materials

The following supporting information can be downloaded at: https://www.mdpi.com/article/10.3390/antiox14060677/s1, Table S1: The concentrations of total phenolics (TP), total non-flavonoids (TNF), total flavonoids (TF), and antioxidant capacity (obtained by DPPH, ABTS, and FRAP assays) in *A. altissima* and *H. tuberosus* leaf and flower extracts in two solvents; Table S2: Pearson’s correlation coefficients (two-tailed) between total phenolic (TP), total non-flavonoids (TNF) and total flavonoids (TF) contents, main phenolic groups, and antioxidant capacity (obtained by DPPH, ABTS, and FRAP assays); Table S3 Pearson’s correlation coefficients (two-tailed) between selected individual phenolic compounds and antioxidant capacity (obtained by DPPH, ABTS, and FRAP assays); Table S4 Pearson’s correlation coefficients (two-tailed) between individual phenolic compounds and antioxidant capacity (obtained by DPPH, ABTS, and FRAP assays)*;* Table S5: Phenolic compounds of *Ailanthus altissima* (Mill.) Swingle and *Helianthus tuberosus* L. leaf and flower extracts identified by LC-DAD-MS in two solvents; Table S6: Individual compounds used for PCA analysis.

## References

[1] Uzelac M., Sladonja B., Šola I., Dudaš S., Bilić J., Famuyide I.M., McGaw L.J., Eloff J.N., Mikulic-Petkovsek M., Poljuha D. (2023). Invasive Alien Species as a Potential Source of Phytopharmaceuticals: Phenolic Composition and Antimicrobial and Cytotoxic Activity of *Robinia pseudoacacia* L. Leaf and Flower Extracts. Plants.

[2] Purmalis O., Klavins L., Niedrite E., Mezulis M., Klavins M. (2025). Invasive Plants as a Source of Polyphenols with High Radical Scavenging Activity. Plants.

[3] Kim Y.O., Lee E.J. (2011). Comparison Of Phenolic Compounds And The Effects of Invasive and Native Species in East Asia: Support for The Novel Weapons Hypothesis. Ecol. Res..

[4] Kato-Noguchi H., Kato M. (2022). Allelopathy and Allelochemicals of *Solidago canadensis* L. and *S. altissima* L. for Their Naturalization. Plants.

[5] Batish D.R., Singh H.P., Priyanka S.K., Kohli R.K., Jose S., Singh H.P., Batish D.R., Kohli R.K. (2013). Novel Weapon Hypothesis for the Successful Establishment of Invasive Plants in Alien Environments. Invasive Plant Ecology.

[6] Kumar Rai P., Singh J.S. (2020). Invasive Alien Plant Species: Their Impact on Environment, Ecosystem Services and Human Health. Ecol. Indic..

[7] Demeter A., Saláta D., Tormáné Kovács E., Szirmai O., Trenyik P., Meinhardt S., Rusvai K., Verbényiné Neumann K., Schermann B., Szegleti Z. (2021). Effects of the Invasive Tree Species *Ailanthus altissima* on the Floral Diversity and Soil Properties in the Pannonian Region. Land.

[8] Sladonja B., Sušek M., Guillermic J. (2015). Review on Invasive Tree of Heaven (*Ailanthus altissima* (Mill.) Swingle) Conflicting Values: Assessment of Its Ecosystem Services and Potential Biological Threat. Environ. Manag..

[9] Feret P.P. (1985). *Ailanthus*: Variation, Cultivation, and Frustration. J. Arboric..

[10] Kowarik I., Säumel I. (2007). Biological Flora of Central Europe: Ailanthus Altissima (Mill.) Swingle. Perspect. Plant Ecol. Evol. Syst..

[11] Regulation (EU) 2019/1262 Commission Implementing Regulation (EU) 2019/1262 of 25 July 2019 Amending Implementing Regulation (EU) 2016/1141 to Update the List of Invasive Alien Species of Union Concern. https://eur-lex.europa.eu/legal-content/EN/TXT/?qid=1565100914543&uri=CELEX:32019R1262.

[12] Strane Invazivne Vrste. https://invazivnevrste.haop.hr/karta.

[13] Heisey R.M. (1996). Identification of an Allelopathic Compound from *Ailanthus altissima* (Simaroubaceae) and Characterization of Its Herbicidal Activity. Am. J Bot..

[14] Meng P., Pei H., Hu W., Liu Z., Li X., Xu H. (2015). Allelopathic Effects of *Ailanthus altissima* Extracts on *Microcystis aeruginosa* Growth, Physiological Changes and Microcystins Release. Chemosphere.

[15] Ullah Z. (2020). Allelopathic Effect of *Ailanthus altissima* on Wheat (*Triticum aestivum* L.). Pure Appl. Biol..

[16] Poljuha D., Sladonja B., Šola I., Dudaš S., Bilić J., Rusak G., Motlhatlego K.E., Eloff J.N. (2017). Phenolic Composition of Leaf Extracts of *Ailanthus altissima* (Simaroubaceae) with Antibacterial and Antifungal Activity Equivalent to Standard Antibiotics. Nat. Prod. Commun..

[17] Marinaș I.C., Dinu M., Ancuceanu R., Hovaneț M.V., Oprea E., Geană E.-I., Lazăr V. (2018). The Phenols Content and Phytotoxic Capacity of Various Invasive Plants. Rom. Biotechnol. Lett..

[18] Andonova T.G., Dimitrova-Dyulgerova I.Z., Slavov Z., Dincheva I.N., Stoyanova A.S. (2021). Volatile Compounds in Flowers, Samaras, Leaves and Stem Bark of *Ailanthus altissima* (Mill.) Swingle, Growing in Bulgaria. IOP Conf. Ser. Mater. Sci. Eng..

[19] Mohamed H., El-Wakil E., Abdel-Hameed E.-S., El-Hashash M., Shemis M. (2021). Evaluation of Total Phenolics, Flavonoids, and Antioxidant and Cytotoxic Potential of *Ailanthus altissima* (Mill.) Swingle Leaves. J. Rep. Pharm. Sci..

[20] Poljuha D., Sladonja B., Šola I., Šenica M., Uzelac M., Veberič R., Hudina M., Famuyide I.M., Eloff J.N., Mikulic-Petkovsek M. (2022). LC–DAD–MS Phenolic Characterisation of Six Invasive Plant Species in Croatia and Determination of Their Antimicrobial and Cytotoxic Activity. Plants.

[21] Andonova T., Muhovski Y., Slavov I., Vrancheva R., Georgiev V., Apostolova E., Naimov S., Mladenov R., Pavlov A., Dimitrova-Dyulgerova I. (2023). Phenolic Profile, Antioxidant and DNA-Protective Capacity, and Microscopic Characters of *Ailanthus altissima* Aerial Substances. Plants.

[22] Popay I. (2022). Helianthus tuberosus (Jerusalem artichoke).

[23] Liava V., Karkanis A., Danalatos N., Tsiropoulos N. (2021). Cultivation Practices, Adaptability and Phytochemical Composition of Jerusalem Artichoke (*Helianthus tuberosus* L.): A Weed with Economic Value. Agronomy.

[24] Yang L., He Q.S., Corscadden K., Udenigwe C.C. (2015). The Prospects of Jerusalem Artichoke in Functional Food Ingredients and Bioenergy Production. Biotechnol. Rep..

[25] Showkat M.M., Falck-Ytter A.B., Strætkvern K.O. (2019). Phenolic Acids in Jerusalem Artichoke (*Helianthus tuberosus* L.): Plant Organ Dependent Antioxidant Activity and Optimized Extraction from Leaves. Molecules.

[26] Zhou B., Jin Z., Schwarz P., Li Y. (2020). Impact of Genotype, Environment, and Malting Conditions on the Antioxidant Activity and Phenolic Content in US Malting Barley. Fermentation.

[27] Pérez-Ochoa M.L., Vera-Guzmán A.M., Mondragón-Chaparro D.M., Sandoval-Torres S., Carrillo-Rodríguez J.C., Chávez-Servia J.L. (2022). Effects of Growth Conditions on Phenolic Composition and Antioxidant Activity in the Medicinal Plant *Ageratina petiolaris* (Asteraceae). Diversity.

[28] Griskeviciene U., Dambrauskiene J., Marksa M., Mazeliene Z., Vainoriene R., Ivanauskas L. (2024). Effect of the Phenological Stage on the Phenolic Composition, and Antioxidant and Antimicrobial Properties of *Cirsium vulgare* (Savi) Ten. Extracts. Life.

[29] Cocîrlea M.D., Soare A., Petrovici A.R., Silion M., Călin T., Oancea S. (2024). Phenolic Composition and Bioactivities of Invasive *Ailanthus altissima* (Mill.) Swingle Leaf Extracts Obtained by Two-Step Sequential Extraction. Antioxidants.

[30] Bilić J., Svorcina M., Poljuha D. Antioxidant Capacity of Fruit Species Characteristic for Gardens in Istria (Croatia). Proceedings of the 9th International Congress of Food Technologists, Biotechnologists and Nutritionists.

[31] Mikulic-Petkovsek M., Schmitzer V., Stampar F., Veberic R., Koron D. (2014). Changes in Phenolic Content Induced by Infection with *Didymella Applanata* and *Leptosphaeria Coniothyrium*, the Causal Agents of Raspberry Spur and Cane Blight. Plant Pathol..

[32] Singleton V.L., Rossi J.A. (1965). Colorimetry of Total Phenolics with Phosphomolybdic-Phosphotungstic Acid Reagents. Am. J. Enol. Vitic..

[33] Amerine M.A., Ough C.S. (1981). Methods for Analysis of Musts and Wines. J. Inst. Brew..

[34] Martins D., Barros L., Carvalho A.M., Ferreira I.C.F.R. (2011). Nutritional and In Vitro Antioxidant Properties of Edible Wild Greens in Iberian Peninsula Traditional Diet. Food Chem..

[35] Poljuha D., Šola I., Bilić J., Dudaš S., Bilušić T., Markić J., Rusak G. (2015). Phenolic Composition, Antioxidant Capacity, Energy Content and Gastrointestinal Stability of Croatian Wild Edible Plants. Eur. Food Res. Technol..

[36] Mikulic-Petkovsek M., Koron D., Rusjan D. (2020). The Impact of Food Processing on the Phenolic Content in Products Made from Juneberry (*Amelanchier lamarckii*) Fruits. J. Food Sci..

[37] Mikulic-Petkovsek M., Stampar F., Veberic R., Sircelj H. (2016). Wild *Prunus* Fruit Species as a Rich Source of Bioactive Compounds. J. Food Sci..

[38] Clifford M.N., Knight S., Kuhnert N. (2005). Discriminating between the Six Isomers of Dicaffeoylquinic Acid by LC-MS^n^. J. Agric. Food Chem..

[39] Luís Â., Gil N., Amaral M.E., Domingues F., Duarte A.P. (2012). *Ailanthus altissima* (Miller) Swingle: A Source of Bioactive Compounds with Antioxidant Activity. BioResources.

[40] Sreekanth D., Devi P. (2019). Qualitative and Quantitative Phytochemical Studies of *Helianthus tuberosus* L.. J. Pharmacogn. Phytochem..

[41] Alyas N.D., Zulkifli N., Noor Hasnan N.Z. (2020). Evaluation of Total Phenolic Content and Antioxidant Activities from Different Extraction Techniques of *Helianthus tuberosus*. Asian Aust. Food Res. J..

[42] Chen F., Long X., Liu Z., Shao H., Liu L. (2014). Analysis of Phenolic Acids of Jerusalem Artichoke (*Helianthus tuberosus* L.) Responding to Salt-Stress by Liquid Chromatography/Tandem Mass Spectrometry. Sci. World J..

[43] Do Q.-D., Angkawijaya A.E., Tran-Nguyen P.L., Huynh L.H., Soetaredjo F.E., Ismadji S., Ju Y.-H. (2014). Effect of Extraction Solvent on Total Phenol Content, Total Flavonoid Content, and Antioxidant Activity of *Limnophila aromatica*. J. Food Drug Anal..

[44] Yuan X., Gao M., Xiao H., Tan C., Du Y. (2012). Free Radical Scavenging Activities and Bioactive Substances of Jerusalem Artichoke (*Helianthus tuberosus* L.) Leaves. Food Chem..

[45] US Food and Drug Administration (FDA) Title 21 Code of Federal Regulations (CFR) §184.1293—Ethyl Alcohol. https://www.ecfr.gov/current/title-21/chapter-I/subchapter-B/part-184/subpart-B/section-184.1293.

[46] Poljuha D., Uzelac Božac M., Čondić A., Pavičić I., Sladonja B., Barišić K. (2025). Exploring the Medicinal Potential of Invasive Plants: Impact on Cellular and Extracellular Glutathione-S-Transferase Activity. J. Polytech. Rijeka.

[47] Kaszás L., Alshaal T., El-Ramady H., Kovács Z., Koroknai J., Elhawat N., Nagy É., Cziáky Z., Fári M., Domokos-Szabolcsy É. (2020). Identification of Bioactive Phytochemicals in Leaf Protein Concentrate of Jerusalem Artichoke (*Helianthus tuberosus* L.). Plants.

[48] Peng S., Scalbert A., Monties B. (1991). Insoluble Ellagitannins in *Castanea sativa* and *Quercus petraea* Woods. Phytochemistry.

[49] Auzanneau C., Montaudon D., Jacquet R., Puyo S., Pouységu L., Deffieux D., Elkaoukabi-Chaibi A., De Giorgi F., Ichas F., Quideau S. (2012). The Polyphenolic Ellagitannin Vescalagin Acts as a Preferential Catalytic Inhibitor of the α Isoform of Human DNA Topoisomerase II. Mol. Pharmacol..

[50] Shen S.C., Chang W.C. (2013). Hypotriglyceridemic and Hypoglycemic Effects of Vescalagin from Pink Wax Apple [*Syzygium samarangense* (Blume) Merrill and Perry Cv. Pink] in High-Fructose Diet-Induced Diabetic Rats. Food Chem..

[51] Araújo A.R., Araújo A.C., Reis R.L., Pires R.A. (2021). Vescalagin and Castalagin Present Bactericidal Activity Toward Methicillin-Resistant Bacteria. ACS Biomater. Sci. Eng..

[52] Chen H., Zuo Y., Deng Y. (2001). Separation and Determination of Flavonoids and Other Phenolic Compounds in Cranberry Juice by High-Performance Liquid Chromatography. J. Chromatogr. A.

[53] Lee S., Lee J., Lee H., Sung J. (2019). Relative Protective Activities of Quercetin, Quercetin-3-Glucoside, and Rutin in Alcohol-Induced Liver Injury. J. Food Biochem..

[54] Prakash M., Basava R.B.V., Murthy K.N., Mohithe P.M. (2018). Biological Functions of Epicatechin: Plant Cell to Human Cell Health. J. Funct. Foods.

[55] Qu Z., Liu A., Li P., Liu C., Xiao W., Huang J., Liu Z., Zhang S. (2021). Advances in Physiological Functions and Mechanisms of (−)-Epicatechin. Crit. Rev. Food Sci. Nutr..

[56] Tchoné M., Bärwald G., Annemüller G., Fleischer L. (2006). Séparation et Identification Des Composés Phénoliques Du Topinambour (*Helianthus tuberosus* L.). Sci. Aliments.

[57] Kapusta I., Krok E.S., Jamro D.B., Cebulak T., Kaszuba J., Salach R.T. (2013). Identification and Quantification of Phenolic Compounds from Jerusalem Artichoke (*Helianthus tuberosus* L.) Tubers. J. Food Agric. Environ..

[58] Bach V., Clausen M., Edelenbos M., Yahia E.M. (2019). Production of Jerusalem Artichoke (*Helianthus tuberosus* L.) and Impact on Inulin and Phenolic Compounds. Postharvest Physiology and Biochemistry of Fruits and Vegetables.

[59] Meinhart A.D., Damin F.M., Caldeirão L., de Jesus Filho M., da Silva L.C., da Silva Constant L., Teixeira Filho J., Wagner R., Teixeira Godoy H. (2019). Study of New Sources of Six Chlorogenic Acids and Caffeic Acid. J. Food Compos. Anal..

[60] Huang J., Xie M., He L., Song X., Cao T. (2023). Chlorogenic Acid: A Review on Its Mechanisms of Anti-Inflammation, Disease Treatment, and Related Delivery Systems. Front. Pharmacol..

[61] Song D., Zhang L., Liu Q., Chen J., Wang T., Li X., Wu J. (2023). Research Progress on the Structural and Functional Comparison, Structural Modification of Chlorogenic Acid and Its Isomers and Application in Animals. Chin. J. Anim. Sci..

[62] Zhang Q., Kim H.-Y. (2015). Antioxidant, Anti-Inflammatory and Cytotoxicity on Human Lung Epithelial A549 Cells of Jerusalem Artichoke (*Helianthus tuberosus* L.) Tuber. Korean J. Plant Res..

[63] Jantaharn P., Mongkolthanaruk W., Senawong T., Jogloy S., McCloskey S. (2018). Bioactive Compounds from Organic Extracts of *Helianthus tuberosus* L. Flowers. Ind. Crops Prod..

[64] Zhu W., Cadet P., Neuwirth L.S. (2023). Bioactive Chemicals in *Helianthus tuberosus* L May Reduce Beta-Amyloid Cytotoxicity as a Potential Novel Treatment for Alzheimer’s Disease. Studies in Natural Products Chemistry.

[65] Docampo M., Olubu A., Wang X., Pasinetti G., Dixon R.A. (2017). Glucuronidated Flavonoids in Neurological Protection: Structural Analysis and Approaches for Chemical and Biological Synthesis. J. Agric. Food Chem..

[66] Ho L., Ferruzzi M.G., Janle E.M., Wang J., Gong B., Chen T.Y., Lobo J., Cooper B., Wu Q.L., Talcott S.T. (2013). Identification of Brain Targeted Bioactive Dietary Quercetin-3-O-Glucuronide as a Novel Intervention for Alzheimer’s Disease. FASEB J..

[67] Aruoma O.I. (2003). Methodological Considerations for Characterizing Potential Antioxidant Actions of Bioactive Components in Plant Foods. Mutat. Res..

[68] Luís Â., Domingues F., Domingues P. (2012). Flavonoids as Antioxidants: Mechanisms and Structure-Activity Relationships. Food Chem..

[69] Tapera R.F., Siwe-Noundou X., Shai L.J., Mokhele S. (2024). Exploring the Therapeutic Potential, Ethnomedicinal Values, and Phytochemistry of *Helianthus tuberosus* L.: A Review. Pharmaceuticals.

[70] Tanasković S., Gvozdenac S., Kolarov R., Bursić V., Konstantinović B., Prvulović D. (2021). Antifeeding and Insecticidal Activity of *Ailanthus altissima* and *Morus alba* Extracts against Gypsy Moth (*Lymantria dispar* [L.], Lepidoptera, Lymantridae) Larvae under Laboratory Conditions. J. Entomol. Res. Soc..

