# Supplementary material for: Phytochemical Profile and Antioxidant Properties of Invasive Plants Ailanthus altissima (Mill.) Swingle and Helianthus tuberosus L. in Istria Region, Croatia
Source: Antioxidants (Basel). 2025 Jun 3;14(6):677. doi: 10.3390/antiox14060677 (PMC12189882; doi:10.3390/antiox14060677)
Supplement: Supplementary file 1 [file antioxidants-14-00677-s001.zip › SUPPLEMENTS/Table S1.pdf]

Table S1. The concentrations of total phenolics (TP), total non-flavonoids (TNF), total flavonoids (TF), and antioxidant capacity (obtained by DPPH, ABTS, and FRAP assays) in *Ailanthus altissima* and *Helianthus tuberosus* leaf and flower extracts in two solvents; EtOH-ethanol, MeOH-methanol.

Analysis	***A. altissima***				***H. tuberosus***			
	LEAF		FLOWER		LEAF		FLOWER	
	70% EtOH	80 % MeOH	70% EtOH	80 % MeOH	70% EtOH	80 % MeOH	70% EtOH	80 % MeOH
TP* (mg GAE/g DW)	157.32 ± 4.03 ^{a}	67.58 ± 1.42 ^{c}	118.01 ± 10.91 ^{b}	53.77 ± 1.51 ^{c}	120.71 ± 2.13 ^{A}	52.65 ± 3.65 ^{B}	39.32 ± 2.15 ^{C}	15.82 ± 1.35 ^{D}
TNF (mg GAE/g DW)	116.30 ± 2.49 ^{a}	40.43 ± 8.27 ^{b}	48.50 ± 4.42 ^{b}	22.38 ± 1.94 ^{c}	80.89 ± 3.12 ^{A}	21.10 ± 3.56 ^{C}	34.35 ± 2.15 ^{B}	9.43 ± 1.33 ^{D}
TF (mg CE/g DW)	18.04 ± 0.74 ^{a}	8.07 ± 1.15 ^{b}	17.48 ± 0.33 ^{a}	8.91 ± 0.71 ^{b}	39.03 ± 2.33 ^{A}	15.68 ± 1.05 ^{B}	38.55 ± 1.17 ^{A}	16.48 ± 2.11 ^{B}
ABTS (mg TE/g DW)	166.38 ± 11.76 ^{ab}	109.56 ± 7.88 ^{c}	186.38 ± 10.23 ^{a}	140.78 ± 13.24 ^{b}	65.43 ± 3.91 ^{B}	29.25 ± 1.63 ^{C}	76.87 ± 3.18 ^{A}	25.56 ± 2.96 ^{C}
Phenolics efficiency (TE/GAE)*	1.06	1.62	1.58	2.62	0.54	0.56	1.95	1.62
DPPH (mg TE/g DW)	190.74 ± 2.67 ^{a}	159.64 ± 1.05 ^{a}	170.60 ± 2.09 ^{a}	185.13 ± 27.81 ^{a}	102.54 ± 1.481 ^{A}	38.93 ± 3.75 ^{C}	84.60 ± 1.93 ^{B}	38.48 ± 4.74 ^{C}
Phenolics efficiency (TE/GAE)*	1.21	2.36	1.45	3.44	0.85	0.74	2.51	2.43
FRAP (mg TE/g DW)	81.87 ± 1.27 ^{a}	55.71 ± 0.35 ^{c}	84.6 ± 1.71 ^{a}	65.86 ± 6.95 ^{d}	76.98 ± 1.53 ^{A}	11.03 ± 0.30 ^{B}	75.36 ± 1.63 ^{A}	11.16 ± 0.35 ^{B}
Phenolics efficiency (TE/GAE)*	0.52	0.82	0.72	1.22	0.63	0.21	1.92	0.71

Data are presented as mean ± SD; Mean values within one method and species followed by different letters (a-d; A-D) are significantly different at the 1% level of probability (ANOVA, Tukey test, $p \leq 0.01$), * normalization of total antioxidant capacity to total phenolic content (mg TE/mg GAE ratios).
