# Supplementary material for: Phytochemical Profile and Antioxidant Properties of Invasive Plants Ailanthus altissima (Mill.) Swingle and Helianthus tuberosus L. in Istria Region, Croatia
Source: Antioxidants (Basel). 2025 Jun 3;14(6):677. doi: 10.3390/antiox14060677 (PMC12189882; doi:10.3390/antiox14060677)
Supplement: Supplementary file 1 [file antioxidants-14-00677-s001.zip › SUPPLEMENTS/Table S2.pdf]

Table S2. Pearson's correlation coefficients (Two-Tailed) between total phenolic (TP), total non-flavonoids (TNF) and total flavonoids (TF) contents, main phenolic groups, and antioxidant capacity (obtained by DPPH, ABTS, and FRAP assays).

Correlation	TNF (mg GAE/g DW)	TF (mg CE/g DW)	FRAP (mg TE/g DW)	ABTS (mg TE/g DW)	DPPH (mg TE/g DW)	HCA (mg/g DW)	HBA (mg/g DW)	FLAVANOLS (mg/g DW)	FLAVONOLS (mg/g DW)	FLAVANONES (mg/g DW)	ELAGITANNINS (mg/g DW)	FLAVONES (mg/g DW)
TP (mg GAE/g DW)	0,904**	0,096	0,624**	0,494**	0,618**	0,280	0,508	0,388*	0,698**	0,404*	0,346	0,386*
TNF (mg GAE/g DW)		0,287	0,649**	0,489**	0,508**	0,468**	0,598**	0,148	0,710**	0,136	0,257	0,485**
TF (mg CE/g DW)			0,342	-0,275	-0,334	0,910**	-0,251	-0,627**	0,507**	-0,671**	-0,568**	-0,262
FRAP (mg TE/g DW)				0,769*	0,755**	0,664**	0,560**	0,344	0,915**	0,357*	0,508**	0,511**
ABTS (mg TE/g DW)					0,930**	0,089	0,854**	0,678**	0,560**	0,688**	0,919**	0,848**
DPPH (mg TE/g DW)						0,042	0,721**	0,785**	0,585**	0,827**	0,897**	0,669**
HCA (mg/g DW)							0,011	-0,366*	0,755**	-0,378*	-0,252	-0,034
HBA (mg/g DW)								0,467*	0,408*	0,466**	0,795**	0,986**
FLAVANOLS (mg/g DW)									0,253	0,936**	0,807**	0,477*
FLAVONOLS (mg/g DW)										0,259	0,323	0,322
FLAVANONES (mg/g DW)											0,824**	0,432*
ELAGITANNINS (mg/g DW)												0,808**

**significant correlation at the 1% level of probability ($p \leq 0.01$)

*significant correlation at the 5% level of probability ($p \leq 0.05$)
