# Supplementary material for: Phytochemical Profile and Antioxidant Properties of Invasive Plants Ailanthus altissima (Mill.) Swingle and Helianthus tuberosus L. in Istria Region, Croatia
Source: Antioxidants (Basel). 2025 Jun 3;14(6):677. doi: 10.3390/antiox14060677 (PMC12189882; doi:10.3390/antiox14060677)
Supplement: Supplementary file 1 [file antioxidants-14-00677-s001.zip › SUPPLEMENTS/Table S3.pdf]

Table S3. Pearson's correlation coefficients (Two-Tailed) between selected individual phenolic compounds and antioxidant capacity (obtained by DPPH, ABTS, and FRAP assays).

Species	Compound	FRAP (mg TE/g DW)	ABTS (mg TE/g DW)	DPPH (mg TE/g DW)
Ailanthus altissima	Total hydroxycynamic acid derivatives	0,902**	0,918**	0,823**
	5-caffeoyliqinic acid	0.964**	0.982**	0.889**
	5-caffeoyliqinic acid 2	0.878**	0.804**	0.911**
	4-coumaroyliquinic acid 2	0.963**	0.949**	0.949**
	5-coumaroyliquinic acid 1	0.963**	0.970**	0.898**
	Total hydroxybenzoicacid derivates	0.902**	0.918**	0.823**
	Protocatechuic acid	0.909**	0.932**	0.828**
	Total flavonols	0.981**	0.922**	0.990**
	Quercetin-3-rutin oxide	0.978**	0.937**	0.946**
	Kaempferol-3-rutinoside	0.976**	0.956**	0.936**
	Quercetin-3- glucuronide	0.981**	0.956**	0.964**
	Kaempferol-3-glucuronide	0.997**	0.969**	0.966**
	Quercetin malosylhexoside	-0.961**	-0.946**	-0.937**
Helianthus tuberosus	Total hydroxycynamic acid derivatives	0,922**	0,845**	0,302
	4-caffeoyliqinic acid 1	0.901**	0.769**	0.693**
	Caffeic acid	0.874**	0.744**	0.568**
	Total hydroxybenzoic acid derivatives	0.872**	0.765**	0.235
	Elagic acid	0.892**	0.800**	0.239
	Total flavonols	0.576*	0.440	0.175
	Quercetin-3-glucoside	0.886**	0.872**	0.259
	Quercetin acetyl hexoside 2	0.919**	0.845**	0.233
	Total flavanones	-0.772**	-0.759**	-0.120
	Naringenin hexoside 3	0.866**	0.795**	0.181
	Naringenin hexoside 4	-0.861**	-0.808**	-0.161
	Total ellagitanins	0.640*	0.718**	0.117
	Vescalagin isomer 1	0.751**	0.797**	0.187
	Vescalagin isomer 2	-0.817**	-0.639*	-0.673*
	HHDP galoylhexose	0.810**	0.661**	0.558*
	HHDP digaloylhexos isomer	0.919**	0.861**	0.468
	Total flavones	0.894**	0.864**	0.141
	Naringenin hexoside	0.866**	0.795**	0.181

**significant correlation at the 1% level of probability ($p \leq 0.01$)

*significant correlation at the 5% level of probability ($p \leq 0.05$)
