# Supplementary material for: Phytochemical Profile and Antioxidant Properties of Invasive Plants Ailanthus altissima (Mill.) Swingle and Helianthus tuberosus L. in Istria Region, Croatia
Source: Antioxidants (Basel). 2025 Jun 3;14(6):677. doi: 10.3390/antiox14060677 (PMC12189882; doi:10.3390/antiox14060677)
Supplement: Supplementary file 1 [file antioxidants-14-00677-s001.zip › SUPPLEMENTS/Table S4.pdf]

Table S4. Pearson's correlation coefficients (Two-Tailed) between individual phenolic compounds and antioxidant capacity (obtained by DPPH, ABTS, and FRAP assays) in 1) Ailanthus altissima and 2) Helianthus tuberosus.

Correlations

		3-caffeoylquinic acid 1	3-caffeoylquinic acid 2	p-coumaric acid hexoside 1	caffeic acid hexoside 1	4-caffeoylquinic acid 1	3 p-coumaroylquinic acid	5-caffeoylquinic acid 1	3-feruloylquinic acid	4-caffeoylquinic acid 2	caffeic acid hexoside 2	5-caffeoylquinic acid 2	caffeic acid	4-feruloylquinic acid	4-p-coumaroylquinic acid 1
3-caffeoylquinic acid 1	Pearson Correlation	1	-,783	-,210	-,026	-,139	-,270	-,503	,308	-,366	,360	,668	-,306	,229	-,652
	Sig. (2-tailed)		,021	,513	,935	,637	,372	,079	,305	,198	,227	,070	,287	,474	,016
	N	14	8	12	12	14	13	13	13	14	13	8	14	12	13
3-caffeoylquinic acid 2	Pearson Correlation	-,783	1	-,447	-,727	-,397	-,287	,728	,290	,127	-,626	-,769	,319	,986	,976
	Sig. (2-tailed)	,021		,374	,101	,330	,532	,064	,528	,764	,132	,026	,442	,000	,000
	N	8	8	6	6	8	7	7	7	8	7	8	8	6	7
p-coumaric acid hexoside 1	Pearson Correlation	-,210	-,447	1	,969	,936	,840	,842	-,423	,794	-,627	,642	,784	-,739	,636
	Sig. (2-tailed)	,513	,374		,000	,000	,001	,001	,171	,001	,029	,169	,002	,009	,026
	N	12	6	13	12	13	12	12	12	13	12	6	13	11	12
caffeic acid hexoside 1	Pearson Correlation	-,026	-,727	,969	1	,996	,964	,941	-,630	,857	-,798	,583	,865	-,878	,543
	Sig. (2-tailed)	,935	,101	,000		,000	,000	,000	,028	,000	,001	,225	,000	,000	,055
	N	12	6	12	13	13	12	13	12	13	13	6	13	12	13
4-caffeoylquinic acid 1	Pearson Correlation	-,139	-,397	,936	,996	1	,983	,876	-,679	,880	-,858	,321	,890	-,920	,584
	Sig. (2-tailed)	,637	,330	,000	,000		,000	,000	,008	,000	,000	,438	,000	,000	,028
	N	14	8	13	13	15	14	14	14	15	14	8	15	13	14
3 p-coumaroylquinic acid	Pearson Correlation	-,270	-,287	,840	,964	,983	1	,882	-,743	,923	-,911	,289	,939	-,954	,616
	Sig. (2-tailed)	,372	,532	,001	,000	,000		,000	,004	,000	,000	,529	,000	,000	,025
	N	13	7	12	12	14	15	13	13	15	13	7	14	12	13
5-caffeoylquinic acid 1	Pearson Correlation	-,503	,728	,842	,941	,876	,882	1	-,567	,912	-,815	-,324	,874	-,889	,851
	Sig. (2-tailed)	,079	,064	,001	,000	,000	,000		,043	,000	,000	,478	,000	,000	,000
	N	13	7	12	13	14	13	14	13	14	14	7	14	13	14
3-feruloylquinic acid	Pearson Correlation	,308	,290	-,423	-,630	-,679	-,743	-,567	1	-,670	,868	,083	-,696	,719	-,300
	Sig. (2-tailed)	,305	,528	,171	,028	,008	,004	,043		,009	,000	,860	,006	,008	,319
	N	13	7	12	12	14	13	13	14	14	13	7	14	12	13
4-caffeoylquinic acid 2	Pearson Correlation	-,366	,127	,794	,857	,880	,923	,912	-,670	1	-,915	,277	,925	-,963	,688
	Sig. (2-tailed)	,198	,764	,001	,000	,000	,000	,000	,009		,000	,506	,000	,000	,007
	N	14	8	13	13	15	15	14	14	16	14	8	15	13	14
caffeic acid hexoside 2	Pearson Correlation	,360	-,626	-,627	-,798	-,858	-,911	-,815	,868	-,915	1	,922	-,926	,953	-,542
	Sig. (2-tailed)	,227	,132	,029	,001	,000	,000	,000	,000	,000		,003	,000	,000	,045
	N	13	7	12	13	14	13	14	13	14	14	7	14	13	14
5-caffeoylquinic acid 2	Pearson Correlation	,668	-,769	,642	,583	,321	,289	-,324	,083	,277	,922	1	,154	-,755	-,641
	Sig. (2-tailed)	,070	,026	,169	,225	,438	,529	,478	,860	,506	,003		,716	,083	,120
	N	8	8	6	6	8	7	7	7	8	7	8	8	6	7
caffeic acid	Pearson Correlation	-,306	,319	,784	,865	,890	,939	,874	-,696	,925	-,926	,154	1	-,949	,654
	Sig. (2-tailed)	,287	,442	,002	,000	,000	,000	,000	,006	,000	,000	,716		,000	,011
	N	14	8	13	13	15	14	14	14	15	14	8	15	13	14
4-feruloylquinic acid	Pearson Correlation	,229	,986	-,739	-,878	-,920	-,954	-,889	,719	-,963	,953	-,755	-,949	1	-,626
	Sig. (2-tailed)	,474	,000	,009	,000	,000	,000	,000	,008	,000	,000	,083	,000		,022
	N	12	6	11	12	13	12	13	12	13	13	6	13	13	13
4-p-coumaroylquinic acid 1	Pearson Correlation	-,652	,976	,636	,543	,584	,616	,851	-,300	,688	-,542	-,641	,654	-,626	1
	Sig. (2-tailed)	,016	,000	,026	,055	,028	,025	,000	,319	,007	,045	,120	,011	,022	
	N	13	7	12	13	14	13	14	13	14	14	7	14	13	14
5- p-coumaroylquinic acid 1	Pearson Correlation	-,088	,306	,257	,232	,200	,157	,302	,305	,084	,183	-,585	-,083	-,066	,624
	Sig. (2-tailed)	,786	,555	,446	,469	,513	,627	,317	,335	,785	,584	,223	,788	,830	,023
	N	12	6	11	12	13	12	13	12	13	13	6	13	13	13

		5- p-coumaroylquinic acid 1	5-feruloylquinic acid 1	Total hydroxy cinnamic acid derivatives	gallic acid	protocatechuic acid	ellagic acid pentoside 1	ellagic acid pentoside 2	ellagic acid	Total hydroxy benzoic acid derivatives	gallocatechin	catechin	procyanidin dimer 1	procyanidin dimer 2	epicatechin	Total flavanols	quercetin galloylhexoside 1	quercetin-3-rutinoside
3-caffeoylquinic acid 1	Pearson Correlation	-,088	,330	-,333	,327	,300	-,123	,331	-,309	-,235	,458	,396	,389	,510	,340	,642	,242	-,211
	Sig. (2-tailed)	,786	,271	,245	,300	,343	,719	,269	,328	,462	,157	,180	,188	,197	,234	,013	,425	,616
	N	12	13	14	12	12	11	13	12	12	11	13	13	8	14	14	13	8
3-caffeoylquinic acid 2	Pearson Correlation	,306	,749	-,069	,467	-,889	-,337	,375	,358	-,344	-,822	-,288	-,317	,039	,960	-,729	-,031	,162
	Sig. (2-tailed)	,555	,053	,871	,351	,018	,514	,408	,486	,504	,045	,531	,488	,927	,000	,040	,948	,702
	N	6	7	8	6	6	6	7	6	6	6	7	7	8	8	8	7	8
p-coumaric acid hexoside 1	Pearson Correlation	,257	-,627	,868	-,550	-,830	,827	-,791	,819	,793	,778	-,447	-,499	,339	-,749	-,064	-,640	-,215
	Sig. (2-tailed)	,446	,029	,000	,064	,002	,003	,002	,001	,002	,008	,145	,099	,511	,003	,835	,025	,683
	N	11	12	13	12	11	10	12	12	12	10	12	12	6	13	13	12	6
caffeic acid hexoside 1	Pearson Correlation	,232	-,797	,923	-,705	-,907	,799	-,910	,893	,885	,784	-,639	-,696	,466	-,879	-,130	-,614	-,361
	Sig. (2-tailed)	,469	,001	,000	,007	,000	,003	,000	,000	,000	,004	,025	,012	,351	,000	,673	,026	,482
	N	12	13	13	13	12	11	12	13	13	11	12	12	6	13	13	13	6
4-caffeoylquinic acid 1	Pearson Correlation	,200	-,855	,933	-,754	-,932	,822	-,926	,921	,918	,761	-,722	-,771	,589	-,910	-,289	-,622	-,299
	Sig. (2-tailed)	,513	,000	,000	,003	,000	,001	,000	,000	,000	,006	,004	,001	,126	,000	,297	,018	,472
	N	13	14	15	13	13	12	14	13	13	11	14	14	8	15	15	14	8
3 p-coumaroylquinic acid	Pearson Correlation	,157	-,910	,947	-,803	-,947	,839	-,950	,947	,945	,680	-,802	-,845	,756	-,951	-,473	-,525	-,331
	Sig. (2-tailed)	,627	,000	,000	,002	,000	,001	,000	,000	,000	,031	,001	,000	,050	,000	,075	,054	,468
	N	12	13	15	12	12	12	14	13	13	10	14	14	7	14	15	14	7
5-caffeoylquinic acid 1	Pearson Correlation	,302	-,799	,954	-,681	-,929	,861	-,921	,941	,902	,445	-,655	-,699	-,331	-,897	-,476	-,693	-,036
	Sig. (2-tailed)	,317	,001	,000	,010	,000	,000	,000	,000	,000	,170	,015	,008	,468	,000	,086	,006	,939
	N	13	14	14	13	13	12	13	13	13	11	13	13	7	14	14	14	7
3-feruloylquinic acid	Pearson Correlation	,305	,850	-,660	,932	,645	-,407	,750	-,670	-,651	-,425	,869	,872	,617	,755	,474	,186	.c
	Sig. (2-tailed)	,335	,000	,010	,000	,023	,215	,002	,017	,022	,221	,000	,000	,140	,002	,087	,543	,000
	N	12	13	14	12	12	11	14	12	12	10	13	13	7	14	14	13	7
4-caffeoylquinic acid 2	Pearson Correlation	,084	-,912	,979	-,797	-,951	,893	-,971	,988	,988	,574	-,836	-,866	,044	-,969	-,488	-,487	-,032
	Sig. (2-tailed)	,785	,000	,000	,001	,000	,000	,000	,000	,000	,065	,000	,000	,917	,000	,055	,065	,940
	N	13	14	16	13	13	13	15	14	14	11	14	14	8	15	16	15	8

		quercetin galloylhexoside 2	kaempferol rhamnosylhexoside 1	quercetin-3-galactoside	kaempferol hexoside 1	quercetin-3-glucoside	kaempferol rhamnosylhexoside 2	quercetin pentoside 1	kaempferol galloylhexoside	quercetin pentoside 2	kaempferol hexoside 2	quercetin acetylhexoside 1	quercetin-3-rhamnoside	kaempferol acetylhexoside 1	quercetin acetylhexoside 2	kaempferol acetylhexoside 2	Total flavonols
3-caffeoylquinic acid 1	Pearson Correlation	-,199	,299	,290	,339	-,195	,152	,724	-,468	,322	-,468	-,353	,362	,241	,105	-,173	,112
	Sig. (2-tailed)	,515	,298	,337	,258	,543	,619	,008	,091	,261	,107	,236	,203	,406	,721	,572	,716
	N	13	14	13	13	12	13	12	14	14	13	13	14	14	14	13	13
3-caffeoylquinic acid 2	Pearson Correlation	,394	,088	,366	,368	-,163	,846	-,774	,824	,795	,610	,374	,519	,229	,888	,151	-,712
	Sig. (2-tailed)	,382	,837	,420	,417	,758	,016	,071	,012	,018	,146	,408	,187	,585	,005	,747	,072
	N	7	8	7	7	6	7	6	8	8	7	7	8	8	8	7	7
p-coumaric acid hexoside 1	Pearson Correlation	,268	-,463	-,554	-,726	,838	-,732	,128	,734	-,620	,685	,836	-,752	-,776	-,630	,760	,596
	Sig. (2-tailed)	,400	,111	,062	,008	,001	,007	,708	,004	,024	,014	,001	,003	,002	,021	,004	,041
	N	12	13	12	12	11	12	11	13	13	12	12	13	13	13	12	12
caffeic acid hexoside 1	Pearson Correlation	,345	-,670	-,649	-,814	,899	-,851	,263	,739	-,801	,805	,891	-,889	-,802	-,552	,662	,650
	Sig. (2-tailed)	,272	,012	,016	,001	,000	,000	,409	,004	,001	,002	,000	,000	,001	,051	,014	,016
	N	12	13	13	13	12	12	12	13	13	12	13	13	13	13	13	13
4-caffeoylquinic acid 1	Pearson Correlation	,385	-,744	-,655	-,832	,931	-,868	,075	,763	-,831	,865	,924	-,914	-,791	-,502	,667	,709
	Sig. (2-tailed)	,174	,001	,011	,000	,000	,000	,809	,001	,000	,000	,000	,000	,000	,056	,009	,004
	N	14	15	14	14	13	14	13	15	15	14	14	15	14	15	14	14
3 p-coumaroylquinic acid	Pearson Correlation	,315	-,781	-,654	-,834	,943	-,879	-,029	,782	-,870	,891	,949	-,938	-,771	-,382	,608	,649
	Sig. (2-tailed)	,273	,001	,011	,000	,000	,000	,926	,001	,000	,000	,000	,000	,001	,160	,021	,012
	N	14	15	14	14	13	14	13	15	15	15	14	15	15	15	14	14
5-caffeoylquinic acid 1	Pearson Correlation	,585	-,671	-,663	-,840	,919	-,740	-,263	,894	-,814	,858	,948	-,905	-,773	-,486	,675	,534
	Sig. (2-tailed)	,032	,009	,010	,000	,000	,004	,385	,000	,000	,000	,000	,000	,001	,078	,008	,049
	N	13	14	14	14	13	13	13	14	14	14	14	14	14	14	14	14
3-feruloylquinic acid	Pearson Correlation	-,136	,880	,359	,539	-,634	,800	-,170	-,491	,751	-,749	-,672	,794	,509	,194	-,331	-,473
	Sig. (2-tailed)	,642	,000	,228	,058	,027	,001	,598	,075	,002	,003	,012	,001	,063	,507	,269	,103
	N	14	14	13	13	12	13	12	14	14	13	13	14	14	14	13	13
4-caffeoylquinic acid 2	Pearson Correlation	,398	-,826	-,620	-,835	,982	-,849	,080	,845	-,884	,917	,986	-,967	-,751	-,383	,672	,694
	Sig. (2-tailed)	,141	,000	,014	,000	,000	,000	,786	,000	,000	,000	,000	,000	,001	,143	,006	,004
	N	15	16	15	15	14	15	14	16	16	15	15	16	16	16	15	15

		naringenin hexoside 1	naringenin hexoside 2	naringenin hexoside 3	naringenin hexoside 4	Total flavanones	vescalagin isomer 1	vescalagin isomer 2	HHDP galloylhexose	HHDP digalloylhexose isomer	Total ellagitannins	apigenin hexoside	Total flavones	TOTAL	FRAP	ABTS	DPPH
3-caffeoylquinic acid 1	Pearson Correlation	,303	-,066	-,399	,464	,518	-,672	-,038	-,121	-,495	-,647	-,513	-,513	-,593	-,449	-,589	,137
	Sig. (2-tailed)	,314	,822	,158	,094	,058	,012	,911	,708	,122	,017	,107	,107	,025	,107	,027	,641
	N	13	14	14	14	14	13	11	12	11	13	11	11	14	14	14	14
3-caffeoylquinic acid 2	Pearson Correlation	-,348	,101	,533	-,010	-,768	,967	,871	,171	,820	,869	,714	,714	,947	,501	,512	-,877
	Sig. (2-tailed)	,445	,812	,173	,982	,026	,000	,055	,747	,089	,011	,111	,111	,000	,206	,194	,004
	N	7	8	8	8	8	7	5	6	5	7	6	6	8	8	8	8
p-coumaric acid hexoside 1	Pearson Correlation	-,517	,753	,818	-,637	-,500	,569	-,618	,629	,706	,497	,904	,904	,733	,825	,758	,773
	Sig. (2-tailed)	,103	,003	,001	,019	,082	,053	,057	,038	,022	,100	,000	,000	,004	,001	,003	,002
	N	11	13	13	13	13	12	10	11	10	12	10	10	13	13	13	13
caffeic acid hexoside 1	Pearson Correlation	-,714	,623	,829	-,783	-,633	,489	-,834	,786	,808	,341	,882	,882	,681	,880	,740	,818
	Sig. (2-tailed)	,014	,023	,000	,002	,020	,090	,001	,002	,005	,255	,001	,001	,010	,000	,004	,001
	N	11	13	13	13	13	13	11	12	10	13	10	10	13	13	13	13
4-caffeoylquinic acid 1	Pearson Correlation	-,767	,533	,869	-,825	-,689	,567	-,869	,839	,861	,452	,913	,913	,737	,901	,769	,683
	Sig. (2-tailed)	,002	,041	,000	,000	,005	,035	,000	,000	,000	,105	,000	,000	,002	,000	,001	,004
	N	13	15	15	15	15	14	12	13	12	14	12	12	15	15	15	15
3 p-coumaroylquinic acid	Pearson Correlation	-,819	,386	,896	-,865	-,760	,626	-,931	,873	,864	,475	,928	,928	,762	,896	,775	,330
	Sig. (2-tailed)	,001	,155	,000	,000	,001	,022	,000	,000	,000	,101	,000	,000	,001	,000	,001	,230
	N	13	15	15	15	15	13	11	12	12	13	13	13	15	15	15	15
5-caffeoylquinic acid 1	Pearson Correlation	-,802	,638	,934	-,855	-,767	,867	-,726	,791	,950	,772	,966	,966	,957	,944	,903	,496
	Sig. (2-tailed)	,002	,014	,000	,000	,001	,000	,007	,001	,000	,001	,000	,000	,000	,000	,000	,071
	N	12	14	14	14	14	14	12	13	11	14	11	11	14	14	14	14
3-feruloylquinic acid	Pearson Correlation	,476	,124	-,595	,853	,670	-,288	,736	-,656	-,637	-,139	-,683	-,683	-,414	-,711	-,647	-,539
	Sig. (2-tailed)	,118	,673	,025	,000	,009	,340	,006	,020	,026	,652	,020	,020	,141	,004	,012	,047
	N	12	14	14	14	14	13	12	12	12	13	11	11	14	14	14	14
4-caffeoylquinic acid 2	Pearson Correlation	-,866	,404	,949	-,920	-,791	,694	-,905	,876	,896	,534	,974	,974	,822	,910	,841	,264
	Sig. (2-tailed)	,000	,121	,000	,000	,000	,006	,000	,000	,000	,049	,000	,000	,000	,000	,000	,323
	N	14	16	16	16	16	14	12	13	12	14	13	13	16	16	16	16

		3-caffeoylquinic acid 1	3-caffeoylquinic acid 2	p-coumaric acid hexoside 1	caffeic acid hexoside 1	4-caffeoylquinic acid 1	3 p-coumaroylquinic acid	5-caffeoylquinic acid 1	3-feruloylquinic acid	4-caffeoylquinic acid 2
vescalagin isomer 2	Pearson Correlation	-,038	,871	-,618	-,834	-,869	-,931	-,726	,736	-,905
	Sig. (2-tailed)	,911	,055	,057	,001	,000	,000	,007	,006	,000
	N	11	5	10	11	12	11	12	12	12
HHDP galloylhexose	Pearson Correlation	-,121	,171	,629	,786	,839	,873	,791	-,656	,876
	Sig. (2-tailed)	,708	,747	,038	,002	,000	,000	,001	,020	,000
	N	12	6	11	12	13	12	13	12	13
HHDP digalloylhexose isomer	Pearson Correlation	-,495	,820	,706	,808	,861	,864	,950	-,637	,896
	Sig. (2-tailed)	,122	,089	,022	,005	,000	,000	,000	,026	,000
	N	11	5	10	10	12	12	11	12	12
Total ellagitannins	Pearson Correlation	-,647	,869	,497	,341	,452	,475	,772	-,139	,534
	Sig. (2-tailed)	,017	,011	,100	,255	,105	,101	,001	,652	,049
	N	13	7	12	13	14	13	14	13	14
apigenin hexoside	Pearson Correlation	-,513	,714	,904	,882	,913	,928	,966	-,683	,974
	Sig. (2-tailed)	,107	,111	,000	,001	,000	,000	,000	,020	,000
	N	11	6	10	10	12	13	11	11	13
Total flavones	Pearson Correlation	-,513	,714	,904	,882	,913	,928	,966	-,683	,974
	Sig. (2-tailed)	,107	,111	,000	,001	,000	,000	,000	,020	,000
	N	11	6	10	10	12	13	11	11	13
TOTAL	Pearson Correlation	-,593	,947	,733	,681	,737	,762	,957	-,414	,822
	Sig. (2-tailed)	,025	,000	,004	,010	,002	,001	,000	,141	,000
	N	14	8	13	13	15	15	14	14	16
FRAP	Pearson Correlation	-,449	,501	,825	,880	,901	,896	,944	-,711	,910
	Sig. (2-tailed)	,107	,206	,001	,000	,000	,000	,000	,004	,000
	N	14	8	13	13	15	15	14	14	16
ABTS	Pearson Correlation	-,589	,512	,758	,740	,769	,775	,903	-,647	,841
	Sig. (2-tailed)	,027	,194	,003	,004	,001	,001	,000	,012	,000
	N	14	8	13	13	15	15	14	14	16
DPPH	Pearson Correlation	,137	-,877	,773	,818	,693	,330	,496	-,539	,264
	Sig. (2-tailed)	,641	,004	,002	,001	,004	,230	,071	,047	,323
	N	14	8	13	13	15	15	14	14	16

*. Correlation is significant at the 0.05 level (2-tailed).

**. Correlation is significant at the 0.01 level (2-tailed).

c. Cannot be computed because at least one of the variables is constant.

Correlations

		3-caffeoylquinic acid	caffeic acid hexoside 1	3-p-coumaroylquinic acid	5-caffeoylquinic acid 1 (5-CQA1)	4-caffeoylquinic acid 1	3-feruloylquinic acid	caffeic acid hex2	5-caffeoylquinic acid 2 (5-CQA2)	4-p-coumaroylquinic acid 1	caffeic acid	5-p-coumaroylquinic acid 1	5-feruloylquinic acid 1	5-p-coumaroylquinic acid 2	5-feruloylquinic acid 2	4-p-coumaroylquinic acid 2	dicaffeoylquinic acid 1	dicaffeoylquinic acid 2	dicaffeoylquinic acid 3	dicaffeoylquinic acid 4	Total hydroxy cinnamic acid derivatives
3-caffeoylquinic acid	Pearson Correlation	1	,561*	-,713**	-,877**	-,862**	,911**	,344	-,770**	-,647*	,846**	-,888**	-,631**	-,936**	-,229	-,880**	-,316	-,819**	,675**	-,122	-,867**
	Sig. (2-tailed)		,024	,003	,000	,000	,000	,228	,000	,012	,000	,000	,009	,000	,394	,000	,233	,000	,004	,653	,000
	N	16	16	15	16	16	14	14	16	14	14	16	16	15	16	15	16	15	16	16	16
caffeic acid hexoside 1	Pearson Correlation	,561*	1	-,278	-,722**	-,631**	,627*	,425	-,446	-,278	,199	-,674**	-,562*	-,601*	-,136	-,523*	-,080	-,753**	,475	-,490	-,678**
	Sig. (2-tailed)	,024		,316	,002	,009	,016	,130	,083	,336	,495	,004	,023	,018	,616	,045	,770	,001	,063	,054	,004
	N	16	16	15	16	16	14	14	16	14	14	16	16	15	16	15	16	15	16	16	16
3-p-coumaroylquinic acid	Pearson Correlation	-,713**	-,278	1	,621*	,682**	-,754**	,094	,885**	,921**	-,446	,656**	,483	,689**	,258	,809**	,785**	,525*	-,054	,213	,668**
	Sig. (2-tailed)	,003	,316		,014	,005	,003	,760	,000	,000	,127	,008	,068	,006	,354	,000	,001	,044	,848	,446	,006
	N	15	15	15	15	15	13	13	15	13	13	15	15	14	15	14	15	15	15	15	15
5-caffeoylquinic acid 1 (5-CQA1)	Pearson Correlation	-,877**	-,722**	,621*	1	,977**	-,843**	-,192	,819**	,622*	-,664**	,994**	,880**	,958**	,448	,951**	,357	,988**	-,519*	,553*	,989**
	Sig. (2-tailed)	,000	,002	,014		,000	,000	,511	,000	,018	,010	,000	,000	,000	,082	,000	,175	,000	,039	,026	,000
	N	16	16	15	16	16	14	14	16	14	14	16	16	15	16	15	16	15	16	16	16
4-caffeoylquinic acid 1	Pearson Correlation	-,862**	-,631**	,682**	,977**	1	-,803**	-,155	,894**	,631*	-,653*	,986**	,908**	,969**	,532*	,976**	,440	,941**	-,448	,526*	,963**
	Sig. (2-tailed)	,000	,009	,005	,000		,001	,597	,000	,016	,011	,000	,000	,000	,034	,000	,088	,000	,082	,036	,000
	N	16	16	15	16	16	14	14	16	14	14	16	16	15	16	15	16	15	16	16	16
3-feruloylquinic acid	Pearson Correlation	,911**	,627*	-,754**	-,843**	-,803**	1	,312	-,724**	-,711**	,726**	-,861**	-,580*	-,895**	,044	-,836**	-,367	-,800**	,602*	-,060	-,851**
	Sig. (2-tailed)	,000	,016	,003	,000	,001		,324	,003	,009	,007	,000	,030	,000	,880	,000	,196	,001	,023	,838	,000
	N	14	14	13	14	14	14	12	14	12	12	14	14	13	14	13	14	13	14	14	14
caffeic acid hex2	Pearson Correlation	,344	,425	,094	-,192	-,155	,312	1	-,110	,194	,485	-,217	-,052	-,253	,058	-,090	,495	-,027	,734**	,371	-,093
	Sig. (2-tailed)	,228	,130	,760	,511	,597	,324		,709	,507	,079	,456	,859	,404	,845	,759	,072	,929	,003	,192	,751
	N	14	14	13	14	14	12	14	14	14	14	14	14	13	14	14	14	13	14	14	14
5-caffeoylquinic acid 2 (5-CQA2)	Pearson Correlation	-,770**	-,446	,885**	,819**	,894**	-,724**	-,110	1	,742**	-,587*	,856**	,787**	,863**	,520*	,961**	,635**	,722**	-,208	,427	,829**
	Sig. (2-tailed)	,000	,083	,000	,000	,000	,003	,709		,002	,027	,000	,000	,000	,039	,000	,008	,002	,438	,099	,000
	N	16	16	15	16	16	14	14	16	14	14	16	16	15	16	15	16	15	16	16	16
4-p-coumaroylquinic acid 1	Pearson Correlation	-,647*	-,278	,921**	,622*	,631*	-,711**	,194	,742**	1	-,273	,618*	,487	,637*	,340	,729**	,784**	,588*	,043	,259	,681**
	Sig. (2-tailed)	,012	,336	,000	,018	,016	,009	,507	,002		,345	,018	,077	,019	,234	,003	,001	,035	,884	,371	,007
	N	14	14	13	14	14	12	14	14	14	14	14	14	13	14	14	14	13	14	14	14
caffeic acid	Pearson Correlation	,846**	,199	-,446	-,664**	-,653*	,726**	,485	-,587*	-,273	1	-,685**	-,410	-,729**	-,077	-,654*	-,030	-,583*	,827**	,217	-,618*
	Sig. (2-tailed)	,000	,495	,127	,010	,011	,007	,079	,027	,345		,007	,145	,005	,792	,011	,919	,037	,000	,456	,018
	N	14	14	13	14	14	12	14	14	14	14	14	14	13	14	14	14	13	14	14	14
5-p-coumaroylquinic acid 1	Pearson Correlation	-,888**	-,674**	,656**	,994**	,986**	-,861**	-,217	,856**	,618*	-,685**	1	,893**	,973**	,457	,964**	,361	,969**	-,515*	,512*	,983**
	Sig. (2-tailed)	,000	,004	,008	,000	,000	,000	,456	,000	,018	,007		,000	,000	,075	,000	,169	,000	,041	,043	,000
	N	16	16	15	16	16	14	14	16	14	14	16	16	15	16	15	16	15	16	16	16
5-feruloylquinic acid 1	Pearson Correlation	-,631**	-,562*	,483	,880**	,908**	-,580*	-,052	,787**	,487	-,410	,893**	1	,819**	,623**	,856**	,384	,856**	-,227	,693**	,878**
	Sig. (2-tailed)	,009	,023	,068	,000	,000	,030	,859	,000	,077	,145	,000		,000	,010	,000	,142	,000	,398	,003	,000
	N	16	16	15	16	16	14	14	16	14	14	16	16	15	16	15	16	15	16	16	16
5-p-coumaroylquinic acid 2	Pearson Correlation	-,936**	-,601*	,689**	,958**	,969**	-,895**	-,253	,863**	,637*	-,729**	,973**	,819**	1	,402	,972**	,377	,904**	-,570*	,333	,951**
	Sig. (2-tailed)	,000	,018	,006	,000	,000	,000	,404	,000	,019	,005	,000	,000		,137	,000	,166	,000	,026	,224	,000
	N	15	15	14	15	15	13	13	15	13	13	15	15	15	15	14	15	14	15	15	15
5-feruloylquinic acid 2	Pearson Correlation	-,229	-,136	,258	,448	,532*	,044	,058	,520*	,340	-,077	,457	,623**	,402	1	,500	,331	,361	,069	,569*	,438
	Sig. (2-tailed)	,394	,616	,354	,082	,034	,880	,845	,039	,234	,792	,075	,010	,137		,058	,211	,187	,799	,021	,090
	N	16	16	15	16	16	14	14	16	14	14	16	16	15	16	15	16	15	16	16	16
4-p-coumaroylquinic acid 2	Pearson Correlation	-,880**	-,523*	,809**	,951**	,976**	-,836**	-,090	,961**	,729**	-,654*	,964**	,856**	,972**	,500	1	,599*	,894**	-,333	,441	,961**
	Sig. (2-tailed)	,000	,045	,000	,000	,000	,000	,759	,000	,003	,011	,000	,000	,000	,058		,018	,000	,226	,100	,000
	N	15	15	14	15	15	13	14	15	14	14	15	15	14	15	15	15	14	15	15	15
dicaffeoylquinic acid 1	Pearson Correlation	-,316	-,080	,785**	,357	,440	-,367	,495	,635**	,784**	-,030	,361	,384	,377	,331	,599*	1	,344	,430	,413	,447
	Sig. (2-tailed)	,233	,770	,001	,175	,088	,196	,072	,008	,001	,919	,169	,142	,166	,211	,018		,209	,096	,112	,083
	N	16	16	15	16	16	14	14	16	14	14	16	16	15	16	15	16	15	16	16	16
dicaffeoylquinic acid 2	Pearson Correlation	-,819**	-,753**	,525*	,988**	,941**	-,800**	-,027	,722**	,588*	-,583*	,969**	,856**	,904**	,361	,894**	,344	1	-,475	,621*	,979**
	Sig. (2-tailed)	,000	,001	,044	,000	,000	,001	,929	,002	,035	,037	,000	,000	,000	,187	,000	,209		,074	,013	,000
	N	15	15	15	15	15	13	13	15	13	13	15	15	14	15	14	15	15	15	15	15
dicaffeoylquinic acid 3	Pearson Correlation	,675**	,475	-,054	-,519*	-,448	,602*	,734**	-,208	,043	,827**	-,515*	-,227	-,570*	,069	-,333	,430	-,475	1	,236	-,432
	Sig. (2-tailed)	,004	,063	,848	,039	,082	,023	,003	,438	,884	,000	,041	,398	,026	,799	,226	,096	,074		,380	,095
	N	16	16	15	16	16	14	14	16	14	14	16	16	15	16	15	16	15	16	16	16
dicaffeoylquinic acid 4	Pearson Correlation	-,122	-,490	,213	,553*	,526*	-,060	,371	,427	,259	,217	,512*	,693**	,333	,569*	,441	,413	,621*	,236	1	,583*
	Sig. (2-tailed)	,653	,054	,446	,026	,036	,838	,192	,099	,371	,456	,043	,003	,224	,021	,100	,112	,013	,380		,018
	N	16	16	15	16	16	14	14	16	14	14	16	16	15	16	15	16	15	16	16	16
Total hydroxy cinnamic acid derivatives	Pearson Correlation	-,867**	-,678**	,668**	,989**	,963**	-,851**	-,093	,829**	,681**	-,618*	,983**	,878**	,951**	,438	,961**	,447	,979**	-,432	,583*	1
	Sig. (2-tailed)	,000	,004	,006	,000	,000	,000	,751	,000	,007	,018	,000	,000	,000	,090	,000	,083	,000	,095	,018	
	N	16	16	15	16	16	14	14	16	14	14	16	16	15	16	15	16	15	16	16	16
kaempferol hexoside 2	Pearson Correlation	-,697**	-,332	,988**	,611*	,681**	-,729**	,048	,894**	,883**	-,442	,639*	,480	,673*	,245	,816**	,819**	,508	-,119	,204	,655*
	Sig. (2-tailed)	,006	,246	,000	,020	,007	,007	,882	,000	,000	,150	,014	,082	,012	,399	,001	,000	,063	,684	,485	,011
	N	14	14	14	14	14	12	12	14	12	12	14	14	13	14	13	14	14	14	14	14
kaempferol-3-glucuronide	Pearson Correlation	-,907**	-,656*	,769**	,968**	,951**	-,890**	-,078	,868**	,763**	-,595*	,972**	,802**	,947**	,320	,955**	,495	,938**	-,459	,474	,976**
	Sig. (2-tailed)	,000	,011	,001	,000	,000	,000	,810	,000	,004	,041	,000	,001	,000	,264	,000	,072	,000	,099	,087	,000
	N	14	14	14	14	14	12	12	14	12	12	14	14	13	14	13	14	14	14	14	14
isorhamnetin hexoside	Pearson Correlation	-,501*	-,597*	,338	,747**	,688**	-,623*	,131	,536*	,324	-,316	,729**	,693**	,697**	,158	,656**	,252	,795**	-,284	,619*	,759**
	Sig. (2-tailed)	,048	,015	,218	,001	,003	,017	,656	,032	,259	,271	,001	,003	,004	,559	,008	,346	,000	,286	,011	,001
	N	16	16	15	16	16	14	14	16	14	14	16	16	15	16	15	16	15	16	16	16
isorhamnetin glucuronide	Pearson Correlation	-,828**	-,700**	,623*	,967**	,928**	-,831**	-,090	,796**	,633*	-,575*	,955**	,833**	,944**	,403	,929**	,419	,967**	-,430	,603*	,976**
	Sig. (2-tailed)	,000	,003	,013	,000	,000	,000	,760	,000	,015	,032	,000	,000	,000	,122	,000	,107	,000	,097	,013	,000
	N	16	16	15	16	16	14	14	16	14	14	16	16	15	16	15	16	15	16	16	16
kaempferol acetylhexoside 1	Pearson Correlation	-,754**	-,349	,829**	,729**	,761**	-,699*	-,047	,869**	,832**	-,563	,759**	,657*	,747**	,636*	,899**	,660*	,669*	-,109	,474	,769**
	Sig. (2-tailed)	,002	,221	,000	,003	,002	,011	,884	,000	,001	,057	,002	,011	,003	,014	,000	,010	,012	,711	,087	,001
	N	14	14	13	14	14	12	12	14	12	12	14	14	13	14	13	14	13	14	14	14
quercetin pentoside 1	Pearson Correlation	,941**	,592*	-,709**	-,869**	-,855**	,914**	,391	-,772**	-,656*	,795**	-,876**	-,638**	-,873**	-,193	-,872**	-,373	-,816**	,571*	-,204	-,863**
	Sig. (2-tailed)	,000	,016	,003	,000	,000	,000	,166	,000	,011	,001	,000	,008	,000	,474	,000	,155	,000	,021	,448	,000
	N	16	16	15	16	16	14	14	16	14	14	16	16	15	16	15	16	15	16	16	16
quercetin pentoside 2	Pearson Correlation	,719**	,712**	-,699**	-,890**	-,886**	,722**	,050	-,826**	-,702**	,387	-,877**	-,832**	-,791**	-,417	-,872**	-,584*	-,869**	,193	-,674**	-,904**
	Sig. (2-tailed)	,002	,002	,004	,000	,000	,004	,865	,000	,005	,172	,000	,000	,000	,108	,000	,018	,000	,474	,004	,000
	N	16	16	15	16	16	14	14	16	14	14	16	16	15	16	15	16	15	16	16	16
quercetin malosyl hexoside	Pearson Correlation	,979**	,586*	-,760**	-,922**	-,910**	,932**	,247	-,824**	-,708**	,820**	-,931**	-,698**	-,953**	-,254	-,929**	-,388	-,868**	,619*	-,241	-,918**
	Sig. (2-tailed)	,000	,017	,001	,000	,000	,000	,394	,000	,005	,000	,000	,003	,000	,342	,000	,137	,000	,011	,369	,000
	N	16	16	15	16	16	14	14	16	14	14	16	16	15	16	15	16	15	16	16	16
Total flavonols	Pearson Correlation	-,895**	-,574*	,878**	,909**	,912**	-,880**	-,070	,916**	,802**	-,619*	,918**	,753**	,923**	,381	,957**	,604*	,858**	-,367	,432	,936**
	Sig. (2-tailed)	,000	,020	,000	,000	,000	,000	,813	,000	,001	,018	,000	,001	,000	,145	,000	,013	,000	,162	,095	,000
	N	16	16	15	16	16	14	14	16	14	14	16	16	15	16	15	16	15	16	16	16
gallic acid	Pearson Correlation	,255	-,218	-,130	,065	,083	,418	,119	,051	-,036	,344	,033	,304	-,212	,236	,012	,217	,122	,527	,617*	,076
	Sig. (2-tailed)	,380	,454	,673	,825	,779	,176	,685	,863	,902	,228	,910	,290	,486	,416	,968	,457	,692	,053	,019	,795
	N	14	14	13	14	14	12	14	14	14	14	14	14	13	14	14	14	13	14	14	14
protocatechuic acid	Pearson Correlation	-,869**	-,681**	,605*	,951**	,940**	-,844**	-,413	,809**	,496	-,747**	,966**	,848**	,921**	,413	,892**	,192	,935**	-,641**	,408	,917**
	Sig. (2-tailed)	,000	,004	,017	,000	,000	,000	,143	,000	,071	,002	,000	,000	,000	,112	,000	,476	,000	,007	,117	,000
	N	16	16	15	16	16	14	14	16	14	14	16	16	15	16	15	16	15	16	16	16
Total hydroxy benzoic acid derivatives	Pearson Correlation	-,842**	-,677**	,604*	,947**	,945**	-,815**	-,400	,824**	,491	-,713**	,964**	,875**	,906**	,442	,894**	,220	,933**	-,587*	,451	,915**
	Sig. (2-tailed)	,000	,004	,017	,000	,000	,000	,156	,000	,074	,004	,000	,000	,000	,087	,000	,414	,000	,017	,079	,000
	N	16	16	15	16	16	14	14	16	14	14	16	16	15	16	15	16	15	16	16	16
quercetin-rhamnosylhexoside	Pearson Correlation	-,354	-,242	-,637	,397	,237	-,047	-,107	-,228	-,735	-,729	,210	,192	,149	-,012	,007	-,117	,455	-,286	,544	,390
	Sig. (2-tailed)	,390	,563	,124	,331	,573	,920	,840	,586	,096	,100	,617	,648	,725	,978	,988	,783	,305	,492	,163	,340
	N	8	8	7	8	8	7	6	8	6	6	8	8	8	8	7	8	7	8	8	8
quercetin-3-rutinoside	Pearson Correlation	-,863**	-,647**	,771**	,957**	,932**	-,847**	-,094	,865**	,741**	-,595*	,959**	,830**	,922**	,416	,941**	,467	,936**	-,406	,552*	,975**
	Sig. (2-tailed)	,000	,007	,001	,000	,000	,000	,750	,000	,002	,025	,000	,000	,000	,109	,000	,068	,000	,119	,026	,000
	N	16	16	15	16	16	14	14	16	14	14	16	16	15	16	15	16	15	16	16	16
isoramnetin hexosylpentoside	Pearson Correlation	-,744**	-,620*	,410	,881**	,825**	-,640*	-,062	,633*	,397	-,488	,864**	,819**	,831**	,417	,784**	,177	,920**	-,495	,579*	,894**
	Sig. (2-tailed)	,002	,018	,164	,000	,000	,025	,848	,015	,202	,107	,000	,000	,000	,138	,002	,544	,000	,072	,030	,000
	N	14	14	13	14	14	12	12	14	12	12	14	14	14	14	13	14	13	14	14	14
quercetin-3-galactoside	Pearson Correlation	-,708**	-,226	,975**	,640*	,666**	-,736**	,179	,832**	,891**	-,471	,672**	,524*	,694**	,273	,804**	,709**	,599*	-,084	,246	,712**
	Sig. (2-tailed)	,003	,418	,000	,010	,007	,004	,559	,000	,000	,104	,006	,045	,006	,325	,001	,003	,024	,765	,376	,003
	N	15	15	14	15	15	13	13	15	13	13	15	15	14	15	14	15	14	15	15	15
quercetin-3-glucoside	Pearson Correlation	-,798**	-,709**	,580*	,944**	,901**	-,756**	-,110	,750**	,579*	-,503	,933**	,857**	,893**	,464	,879**	,330	,943**	-,488	,592*	,950**
	Sig. (2-tailed)	,000	,003	,030	,000	,000	,003	,721	,001	,038	,080	,000	,000	,000	,081	,000	,230	,000	,065	,020	,000
	N	15	15	14	15	15	13	13	15	13	13	15	15	14	15	14	15	14	15	15	15
kaempferol rhamnosylhexoside 1	Pearson Correlation	-,656**	-,664**	,449	,848**	,795**	-,595*	-,007	,634*	,427	-,334	,830**	,809**	,781**	,499	,748**	,261	,867**	-,412	,635*	,856**
	Sig. (2-tailed)	,008	,007	,108	,000	,000	,032	,982	,011	,146	,265	,000	,000	,001	,058	,002	,346	,000	,127	,011	,000
	N	15	15	14	15	15	13	13	15	13	13	15	15	14	15	14	15	14	15	15	15
kaempferol-3-rutinoside	Pearson Correlation	-,918**	-,703**	,738**	,962**	,926**	-,900**	-,246	,846**	,683**	-,672**	,964**	,794**	,940**	,349	,933**	,382	,941**	-,522*	,448	,967**
	Sig. (2-tailed)	,000	,002	,002	,000	,000	,000	,397	,000	,007	,009	,000	,000	,000	,185	,000	,145	,000	,038	,082	,000
	N	16	16	15	16	16	14	14	16	14	14	16	16	15	16	15	16	15	16	16	16
quercetin-3-glucuronide	Pearson Correlation	-,932**	-,563*	,787**	,944**	,939**	-,896**	-,067	,875**	,759**	-,655*	,957**	,762**	,967**	,323	,957**	,481	,900**	-,495	,381	,952**
	Sig. (2-tailed)	,000	,036	,001	,000	,000	,000	,837	,000	,004	,021	,000	,002	,000	,260	,000	,082	,000	,072	,179	,000
	N	14	14	14	14	14	12	12	14	12	12	14	14	13	14	13	14	14	14	14	14
kaempferol hexoside 1	Pearson Correlation	-,616*	-,207	,593*	,617*	,630*	-,711*	-,168	,629*	,596	-,620*	,649*	,511	,661*	,071	,657*	,297	,572*	-,374	,134	,628*
	Sig. (2-tailed)	,025	,496	,033	,025	,021	,014	,621	,021	,053	,042	,016	,074	,019	,819	,020	,325	,041	,208	,662	,022
	N	13	13	13	13	13	11	11	13	11	11	13	13	12	13	12	13	13	13	13	13
TOTAL	Pearson Correlation	-,894**	-,680**	,739**	,987**	,976**	-,865**	-,140	,882**	,709**	-,643*	,987**	,860**	,965**	,446	,975**	,468	,957**	-,455	,534*	,990**
	Sig. (2-tailed)	,000	,004	,002	,000	,000	,000	,634	,000	,005	,013	,000	,000	,000	,083	,000	,068	,000	,077	,033	,000
	N	16	16	15	16	16	14	14	16	14	14	16	16	15	16	15	16	15	16	16	16
FRAP	Pearson Correlation	-,915**	-,672**	,796**	,964**	,952**	-,888**	-,127	,878**	,775**	-,646*	,963**	,800**	,951**	,390	,966**	,513*	,926**	-,455	,474	,973**
	Sig. (2-tailed)	,000	,004	,000	,000	,000	,000	,664	,000	,001	,013	,000	,000	,000	,135	,000	,042	,000	,076	,064	,000
	N	16	16	15	16	16	14	14	16	14	14	16	16	15	16	15	16	15	16	16	16
ABTS	Pearson Correlation	-,905**	-,732**	,655**	,982**	,957**	-,857**	-,196	,804**	,662**	-,692**	,970**	,816**	,958**	,399	,949**	,377	,961**	-,571*	,477	,967**
	Sig. (2-tailed)	,000	,001	,008	,000	,000	,000	,501	,000	,010	,006	,000	,000	,000	,126	,000	,150	,000	,021	,062	,000
	N	16	16	15	16	16	14	14	16	14	14	16	16	15	16	15	16	15	16	16	16
DPPH	Pearson Correlation	-,898**	-,553*	,897**	,889**	,901**	-,854**	-,054	,911**	,851**	-,598*	,898**	,739**	,903**	,386	,949**	,634**	,830**	-,340	,399	,916**
	Sig. (2-tailed)	,000	,026	,000	,000	,000	,000	,854	,000	,000	,024	,000	,001	,000	,140	,000	,008	,000	,198	,126	,000
	N	16	16	15	16	16	14	14	16	14	14	16	16	15	16	15	16	15	16	16	16

		kaempferol hexoside 2	kaempferol-3-glucuronide	isorhamnetin hexoside	isorhamnetin glucuronide	kaempferol acetylhexoside 1	quercetin pentoside 1	quercetin pentoside 2	quercetin malosyl hexoside	Total flavonols	gallic acid	protocatechuic acid	Total hydroxy benzoic acid derivatives	quercetin-rhamnosylhexoside	quercetin-3-rutinoside	isoramnetin hexosylpentoside	quercetin-3-galactoside	quercetin-3-glucoside	kaempferol rhamnosylhexoside 1	kaempferol-3-rutinoside	quercetin-3-glucuronide	kaempferol hexoside 1	TOTAL	FRAP	ABTS	DPPH
3-caffeoylquinic acid	Pearson Correlation	-,697**	-,907**	-,501*	-,828**	-,754**	,941**	,719**	,979**	-,895**	,255	-,869**	-,842**	-,354	-,863**	-,744**	-,708**	-,798**	-,656**	-,918**	-,932**	-,616*	-,894**	-,915**	-,905**	-,898**
	Sig. (2-tailed)	,006	,000	,048	,000	,002	,000	,002	,000	,000	,380	,000	,000	,390	,000	,002	,003	,000	,008	,000	,000	,025	,000	,000	,000	,000
	N	14	14	16	16	14	16	16	16	16	14	16	16	8	16	14	15	15	15	16	14	13	16	16	16	16
caffeic acid hexoside 1	Pearson Correlation	-,332	-,656*	-,597*	-,700**	-,349	,592*	,712**	,586*	-,574*	-,218	-,681**	-,677**	-,242	-,647**	-,620*	-,226	-,709**	-,664**	-,703**	-,563*	-,207	-,680**	-,672**	-,732**	-,553*
	Sig. (2-tailed)	,246	,011	,015	,003	,221	,016	,002	,017	,020	,454	,004	,004	,563	,007	,018	,418	,003	,007	,002	,036	,496	,004	,004	,001	,026
	N	14	14	16	16	14	16	16	16	16	14	16	16	8	16	14	15	15	15	16	14	13	16	16	16	16
3-p-coumaroylquinic acid	Pearson Correlation	,988**	,769**	,338	,623*	,829**	-,709**	-,699**	-,760**	,878**	-,130	,605*	,604*	-,637	,771**	,410	,975**	,580*	,449	,738**	,787**	,593*	,739**	,796**	,655**	,897**
	Sig. (2-tailed)	,000	,001	,218	,013	,000	,003	,004	,001	,000	,673	,017	,017	,124	,001	,164	,000	,030	,108	,002	,001	,033	,002	,000	,008	,000
	N	14	14	15	15	13	15	15	15	15	13	15	15	7	15	13	14	14	14	15	14	13	15	15	15	15
5-caffeoylquinic acid 1 (5-CQA1)	Pearson Correlation	,611*	,968**	,747**	,967**	,729**	-,869**	-,890**	-,922**	,909**	,065	,951**	,947**	,397	,957**	,881**	,640*	,944**	,848**	,962**	,944**	,617*	,987**	,964**	,982**	,889**
	Sig. (2-tailed)	,020	,000	,001	,000	,003	,000	,000	,000	,000	,825	,000	,000	,331	,000	,000	,010	,000	,000	,000	,000	,025	,000	,000	,000	,000
	N	14	14	16	16	14	16	16	16	16	14	16	16	8	16	14	15	15	15	16	14	13	16	16	16	16
4-caffeoylquinic acid 1	Pearson Correlation	,681**	,951**	,688**	,928**	,761**	-,855**	-,886**	-,910**	,912**	,083	,940**	,945**	,237	,932**	,825**	,666**	,901**	,795**	,926**	,939**	,630*	,976**	,952**	,957**	,901**
	Sig. (2-tailed)	,007	,000	,003	,000	,002	,000	,000	,000	,000	,779	,000	,000	,573	,000	,000	,007	,000	,000	,000	,000	,021	,000	,000	,000	,000
	N	14	14	16	16	14	16	16	16	16	14	16	16	8	16	14	15	15	15	16	14	13	16	16	16	16
3-feruloylquinic acid	Pearson Correlation	-,729**	-,890**	-,623*	-,831**	-,699*	,914**	,722**	,932**	-,880**	,418	-,844**	-,815**	-,047	-,847**	-,640*	-,736**	-,756**	-,595*	-,900**	-,896**	-,711*	-,865**	-,888**	-,857**	-,854**
	Sig. (2-tailed)	,007	,000	,017	,000	,011	,000	,004	,000	,000	,176	,000	,000	,920	,000	,025	,004	,003	,032	,000	,000	,014	,000	,000	,000	,000
	N	12	12	14	14	12	14	14	14	14	12	14	14	7	14	12	13	13	13	14	12	11	14	14	14	14
caffeic acid hex2	Pearson Correlation	,048	-,078	,131	-,090	-,047	,391	,050	,247	-,070	,119	-,413	-,400	-,107	-,094	-,062	,179	-,110	-,007	-,246	-,067	-,168	-,140	-,127	-,196	-,054
	Sig. (2-tailed)	,882	,810	,656	,760	,884	,166	,865	,394	,813	,685	,143	,156	,840	,750	,848	,559	,721	,982	,397	,837	,621	,634	,664	,501	,854
	N	12	12	14	14	12	14	14	14	14	14	14	14	6	14	12	13	13	13	14	12	11	14	14	14	14
5-caffeoylquinic acid 2 (5-CQA2)	Pearson Correlation	,894**	,868**	,536*	,796**	,869**	-,772**	-,826**	-,824**	,916**	,051	,809**	,824**	-,228	,865**	,633*	,832**	,750**	,634*	,846**	,875**	,629*	,882**	,878**	,804**	,911**
	Sig. (2-tailed)	,000	,000	,032	,000	,000	,000	,000	,000	,000	,863	,000	,000	,586	,000	,015	,000	,001	,011	,000	,000	,021	,000	,000	,000	,000
	N	14	14	16	16	14	16	16	16	16	14	16	16	8	16	14	15	15	15	16	14	13	16	16	16	16
4-p-coumaroylquinic acid 1	Pearson Correlation	,883**	,763**	,324	,633*	,832**	-,656*	-,702**	-,708**	,802**	-,036	,496	,491	-,735	,741**	,397	,891**	,579*	,427	,683**	,759**	,596	,709**	,775**	,662**	,851**
	Sig. (2-tailed)	,000	,004	,259	,015	,001	,011	,005	,005	,001	,902	,071	,074	,096	,002	,202	,000	,038	,146	,007	,004	,053	,005	,001	,010	,000
	N	12	12	14	14	12	14	14	14	14	14	14	14	6	14	12	13	13	13	14	12	11	14	14	14	14
caffeic acid	Pearson Correlation	-,442	-,595*	-,316	-,575*	-,563	,795**	,387	,820**	-,619*	,344	-,747**	-,713**	-,729	-,595*	-,488	-,471	-,503	-,334	-,672**	-,655*	-,620*	-,643*	-,646*	-,692**	-,598*
	Sig. (2-tailed)	,150	,041	,271	,032	,057	,001	,172	,000	,018	,228	,002	,004	,100	,025	,107	,104	,080	,265	,009	,021	,042	,013	,013	,006	,024
	N	12	12	14	14	12	14	14	14	14	14	14	14	6	14	12	13	13	13	14	12	11	14	14	14	14
5-p-coumaroylquinic acid 1	Pearson Correlation	,639*	,972**	,729**	,955**	,759**	-,876**	-,877**	-,931**	,918**	,033	,966**	,964**	,210	,959**	,864**	,672**	,933**	,830**	,964**	,957**	,649*	,987**	,963**	,970**	,898**
	Sig. (2-tailed)	,014	,000	,001	,000	,002	,000	,000	,000	,000	,910	,000	,000	,617	,000	,000	,006	,000	,000	,000	,000	,016	,000	,000	,000	,000
	N	14	14	16	16	14	16	16	16	16	14	16	16	8	16	14	15	15	15	16	14	13	16	16	16	16
5-feruloylquinic acid 1	Pearson Correlation	,480	,802**	,693**	,833**	,657*	-,638**	-,832**	-,698**	,753**	,304	,848**	,875**	,192	,830**	,819**	,524*	,857**	,809**	,794**	,762**	,511	,860**	,800**	,816**	,739**
	Sig. (2-tailed)	,082	,001	,003	,000	,011	,008	,000	,003	,001	,290	,000	,000	,648	,000	,000	,045	,000	,000	,000	,002	,074	,000	,000	,000	,001
	N	14	14	16	16	14	16	16	16	16	14	16	16	8	16	14	15	15	15	16	14	13	16	16	16	16
5-p-coumaroylquinic acid 2	Pearson Correlation	,673*	,947**	,697**	,944**	,747**	-,873**	-,791**	-,953**	,923**	-,212	,921**	,906**	,149	,922**	,831**	,694**	,893**	,781**	,940**	,967**	,661*	,965**	,951**	,958**	,903**
	Sig. (2-tailed)	,012	,000	,004	,000	,003	,000	,000	,000	,000	,486	,000	,000	,725	,000	,000	,006	,000	,001	,000	,000	,019	,000	,000	,000	,000
	N	13	13	15	15	13	15	15	15	15	13	15	15	8	15	14	14	14	14	15	13	12	15	15	15	15
5-feruloylquinic acid 2	Pearson Correlation	,245	,320	,158	,403	,636*	-,193	-,417	-,254	,381	,236	,413	,442	-,012	,416	,417	,273	,464	,499	,349	,323	,071	,446	,390	,399	,386
	Sig. (2-tailed)	,399	,264	,559	,122	,014	,474	,108	,342	,145	,416	,112	,087	,978	,109	,138	,325	,081	,058	,185	,260	,819	,083	,135	,126	,140
	N	14	14	16	16	14	16	16	16	16	14	16	16	8	16	14	15	15	15	16	14	13	16	16	16	16
4-p-coumaroylquinic acid 2	Pearson Correlation	,816**	,955**	,656**	,929**	,899**	-,872**	-,872**	-,929**	,957**	,012	,892**	,894**	,007	,941**	,784**	,804**	,879**	,748**	,933**	,957**	,657*	,975**	,966**	,949**	,949**
	Sig. (2-tailed)	,001	,000	,008	,000	,000	,000	,000	,000	,000	,968	,000	,000	,988	,000	,002	,001	,000	,002	,000	,000	,020	,000	,000	,000	,000
	N	13	13	15	15	13	15	15	15	15	14	15	15	7	15	13	14	14	14	15	13	12	15	15	15	15
dicaffeoylquinic acid 1	Pearson Correlation	,819**	,495	,252	,419	,660*	-,373	-,584*	-,388	,604*	,217	,192	,220	-,117	,467	,177	,709**	,330	,261	,382	,481	,297	,468	,513*	,377	,634**
	Sig. (2-tailed)	,000	,072	,346	,107	,010	,155	,018	,137	,013	,457	,476	,414	,783	,068	,544	,003	,230	,346	,145	,082	,325	,068	,042	,150	,008
	N	14	14	16	16	14	16	16	16	16	14	16	16	8	16	14	15	15	15	16	14	13	16	16	16	16
dicaffeoylquinic acid 2	Pearson Correlation	,508	,938**	,795**	,967**	,669*	-,816**	-,869**	-,868**	,858**	,122	,935**	,933**	,455	,936**	,920**	,599*	,943**	,867**	,941**	,900**	,572*	,957**	,926**	,961**	,830**
	Sig. (2-tailed)	,063	,000	,000	,000	,012	,000	,000	,000	,000	,692	,000	,000	,305	,000	,000	,024	,000	,000	,000	,000	,041	,000	,000	,000	,000
	N	14	14	15	15	13	15	15	15	15	13	15	15	7	15	13	14	14	14	15	14	13	15	15	15	15
dicaffeoylquinic acid 3	Pearson Correlation	-,119	-,459	-,284	-,430	-,109	,571*	,193	,619*	-,367	,527	-,641**	-,587*	-,286	-,406	-,495	-,084	-,488	-,412	-,522*	-,495	-,374	-,455	-,455	-,571*	-,340
	Sig. (2-tailed)	,684	,099	,286	,097	,711	,021	,474	,011	,162	,053	,007	,017	,492	,119	,072	,765	,065	,127	,038	,072	,208	,077	,076	,021	,198
	N	14	14	16	16	14	16	16	16	16	14	16	16	8	16	14	15	15	15	16	14	13	16	16	16	16
dicaffeoylquinic acid 4	Pearson Correlation	,204	,474	,619*	,603*	,474	-,204	-,674**	-,241	,432	,617*	,408	,451	,544	,552*	,579*	,246	,592*	,635*	,448	,381	,134	,534*	,474	,477	,399
	Sig. (2-tailed)	,485	,087	,011	,013	,087	,448	,004	,369	,095	,019	,117	,079	,163	,026	,030	,376	,020	,011	,082	,179	,662	,033	,064	,062	,126
	N	14	14	16	16	14	16	16	16	16	14	16	16	8	16	14	15	15	15	16	14	13	16	16	16	16
Total hydroxy cinnamic acid derivatives	Pearson Correlation	,655*	,976**	,759**	,976**	,769**	-,863**	-,904**	-,918**	,936**	,076	,917**	,915**	,390	,975**	,894**	,712**	,950**	,856**	,967**	,952**	,628*	,990**	,973**	,967**	,916**
	Sig. (2-tailed)	,011	,000	,001	,000	,001	,000	,000	,000	,000	,795	,000	,000	,340	,000	,000	,003	,000	,000	,000	,000	,022	,000	,000	,000	,000
	N	14	14	16	16	14	16	16	16	16	14	16	16	8	16	14	15	15	15	16	14	13	16	16	16	16
kaempferol hexoside 2	Pearson Correlation	1	,765**	,301	,604*	,819**	-,699**	-,703**	-,748**	,874**	-,083	,612*	,614*	-,407	,738**	,393	,962**	,630*	,523	,704**	,768**	,524	,729**	,793**	,671**	,893**
	Sig. (2-tailed)		,002	,295	,022	,001	,005	,005	,002	,000	,797	,020	,020	,423	,003	,207	,000	,021	,067	,005	,002	,080	,003	,001	,009	,000
	N	14	13	14	14	12	14	14	14	14	12	14	14	6	14	12	13	13	13	14	13	12	14	14	14	14
kaempferol-3-glucuronide	Pearson Correlation	,765**	1	,704**	,947**	,811**	-,890**	-,879**	-,958**	,984**	-,067	,948**	,939**	,121	,991**	,846**	,820**	,931**	,822**	,990**	,985**	,680*	,992**	,997**	,969**	,966**
	Sig. (2-tailed)	,002		,005	,000	,001	,000	,000	,000	,000	,835	,000	,000	,820	,000	,000	,001	,000	,000	,000	,000	,011	,000	,000	,000	,000
	N	13	14	14	14	13	14	14	14	14	12	14	14	6	14	13	13	14	14	14	14	13	14	14	14	14
isorhamnetin hexoside	Pearson Correlation	,301	,704**	1	,832**	,419	-,481	-,605*	-,622*	,644**	-,046	,658**	,643**	,549	,707**	,773**	,407	,768**	,761**	,705**	,689**	,666*	,723**	,679**	,736**	,571*
	Sig. (2-tailed)	,295	,005		,000	,136	,059	,013	,010	,007	,876	,006	,007	,159	,002	,001	,132	,001	,001	,002	,006	,013	,002	,004	,001	,021
	N	14	14	16	16	14	16	16	16	16	14	16	16	8	16	14	15	15	15	16	14	13	16	16	16	16
isorhamnetin glucuronide	Pearson Correlation	,604*	,947**	,832**	1	,712**	-,811**	-,858**	-,886**	,909**	,038	,884**	,877**	,546	,943**	,907**	,663**	,943**	,869**	,951**	,928**	,618*	,965**	,945**	,959**	,874**
	Sig. (2-tailed)	,022	,000	,000		,004	,000	,000	,000	,000	,899	,000	,000	,162	,000	,000	,007	,000	,000	,000	,000	,025	,000	,000	,000	,000
	N	14	14	16	16	14	16	16	16	16	14	16	16	8	16	14	15	15	15	16	14	13	16	16	16	16
kaempferol acetylhexoside 1	Pearson Correlation	,819**	,811**	,419	,712**	1	-,800**	-,758**	-,796**	,867**	,056	,687**	,694**	-,437	,831**	,566*	,866**	,655*	,550*	,797**	,813**	,613*	,799**	,816**	,678**	,874**
	Sig. (2-tailed)	,001	,001	,136	,004		,001	,002	,001	,000	,863	,007	,006	,327	,000	,044	,000	,011	,041	,001	,001	,034	,001	,000	,008	,000
	N	12	13	14	14	14	14	14	14	14	12	14	14	7	14	13	13	13	14	14	13	12	14	14	14	14
quercetin pentoside 1	Pearson Correlation	-,699**	-,890**	-,481	-,811**	-,800**	1	,818**	,939**	-,875**	,021	-,855**	-,848**	.c	-,856**	-,604*	-,693**	-,722**	-,537*	-,890**	-,876**	-,652*	-,884**	-,906**	-,869**	-,881**
	Sig. (2-tailed)	,005	,000	,059	,000	,001		,000	,000	,000	,942	,000	,000	,000	,000	,022	,004	,002	,039	,000	,000	,016	,000	,000	,000	,000
	N	14	14	16	16	14	16	16	16	16	14	16	16	8	16	14	15	15	15	16	14	13	16	16	16	16
quercetin pentoside 2	Pearson Correlation	-,703**	-,879**	-,605*	-,858**	-,758**	,818**	1	,784**	-,869**	-,417	-,806**	-,835**	.c	-,902**	-,679**	-,706**	-,806**	-,684**	-,860**	-,799**	-,501	-,909**	-,906**	-,854**	-,877**
	Sig. (2-tailed)	,005	,000	,013	,000	,002	,000		,000	,000	,138	,000	,000	,000	,000	,008	,003	,000	,005	,000	,001	,081	,000	,000	,000	,000
	N	14	14	16	16	14	16	16	16	16	14	16	16	8	16	14	15	15	15	16	14	13	16	16	16	16
quercetin malosyl hexoside	Pearson Correlation	-,748**	-,958**	-,622*	-,886**	-,796**	,939**	,784**	1	-,944**	,214	-,903**	-,879**	.c	-,920**	-,767**	-,757**	-,853**	-,713**	-,949**	-,975**	-,731**	-,944**	-,961**	-,946**	-,937**
	Sig. (2-tailed)	,002	,000	,010	,000	,001	,000	,000		,000	,462	,000	,000	,000	,000	,001	,001	,000	,003	,000	,000	,005	,000	,000	,000	,000
	N	14	14	16	16	14	16	16	16	16	14	16	16	8	16	14	15	15	15	16	14	13	16	16	16	16
Total flavonols	Pearson Correlation	,874**	,984**	,644**	,909**	,867**	-,875**	-,869**	-,944**	1	-,071	,857**	,848**	-,288	,965**	,784**	,883**	,891**	,775**	,958**	,983**	,692**	,964**	,981**	,922**	,990**
	Sig. (2-tailed)	,000	,000	,007	,000	,000	,000	,000	,000		,810	,000	,000	,489	,000	,001	,000	,000	,001	,000	,000	,009	,000	,000	,000	,000
	N	14	14	16	16	14	16	16	16	16	14	16	16	8	16	14	15	15	15	16	14	13	16	16	16	16
gallic acid	Pearson Correlation	-,083	-,067	-,046	,038	,056	,021	-,417	,214	-,071	1	-,018	,075	,785	,025	-,110	-,202	-,049	-,070	-,065	-,214	-,243	,033	-,012	-,046	-,030
	Sig. (2-tailed)	,797	,835	,876	,899	,863	,942	,138	,462	,810		,950	,799	,065	,932	,734	,508	,875	,820	,825	,505	,471	,912	,966	,876	,920
	N	12	12	14	14	12	14	14	14	14	14	14	14	6	14	12	13	13	13	14	12	11	14	14	14	14
protocatechuic acid	Pearson Correlation	,612*	,948**	,658**	,884**	,687**	-,855**	-,806**	-,903**	,857**	-,018	1	,995**	,134	,910**	,804**	,579*	,901**	,800**	,932**	,930**	,668*	,936**	,909**	,932**	,828**
	Sig. (2-tailed)	,020	,000	,006	,000	,007	,000	,000	,000	,000	,950		,000	,751	,000	,001	,024	,000	,000	,000	,000	,013	,000	,000	,000	,000
	N	14	14	16	16	14	16	16	16	16	14	16	16	8	16	14	15	15	15	16	14	13	16	16	16	16
Total hydroxy benzoic acid derivatives	Pearson Correlation	,614*	,939**	,643**	,877**	,694**	-,848**	-,835**	-,879**	,848**	,075	,995**	1	,144	,906**	,787**	,571*	,887**	,783**	,919**	,909**	,646*	,932**	,902**	,918**	,823**
	Sig. (2-tailed)	,020	,000	,007	,000	,006	,000	,000	,000	,000	,799	,000		,734	,000	,001	,026	,000	,001	,000	,000	,017	,000	,000	,000	,000
	N	14	14	16	16	14	16	16	16	16	14	16	16	8	16	14	15	15	15	16	14	13	16	16	16	16
quercetin-rhamnosylhexoside	Pearson Correlation	-,407	,121	,549	,546	-,437	.c	.c	.c	-,288	,785	,134	,144	1	-,345	,908**	-,592	,805*	,820*	-,028	-,377	-,387	,275	-,505	,542	-,483
	Sig. (2-tailed)	,423	,820	,159	,162	,327	,000	,000	,000	,489	,065	,751	,734		,402	,005	,122	,029	,024	,947	,461	,449	,509	,202	,165	,225
	N	6	6	8	8	7	8	8	8	8	6	8	8	8	8	7	8	7	7	8	6	6	8	8	8	8
quercetin-3-rutinoside	Pearson Correlation	,738**	,991**	,707**	,943**	,831**	-,856**	-,902**	-,920**	,965**	,025	,910**	,906**	-,345	1	,848**	,810**	,932**	,831**	,979**	,969**	,658*	,983**	,978**	,937**	,946**
	Sig. (2-tailed)	,003	,000	,002	,000	,000	,000	,000	,000	,000	,932	,000	,000	,402		,000	,000	,000	,000	,000	,000	,015	,000	,000	,000	,000
	N	14	14	16	16	14	16	16	16	16	14	16	16	8	16	14	15	15	15	16	14	13	16	16	16	16
isoramnetin hexosylpentoside	Pearson Correlation	,393	,846**	,773**	,907**	,566*	-,604*	-,679**	-,767**	,784**	-,110	,804**	,787**	,908**	,848**	1	,516	,943**	,944**	,856**	,840**	,444	,859**	,816**	,869**	,742**
	Sig. (2-tailed)	,207	,000	,001	,000	,044	,022	,008	,001	,001	,734	,001	,001	,005	,000		,071	,000	,000	,000	,000	,148	,000	,000	,000	,002
	N	12	13	14	14	13	14	14	14	14	12	14	14	7	14	14	13	14	14	14	13	12	14	14	14	14
quercetin-3-galactoside	Pearson Correlation	,962**	,820**	,407	,663**	,866**	-,693**	-,706**	-,757**	,883**	-,202	,579*	,571*	-,592	,810**	,516	1	,619*	,502	,769**	,841**	,639*	,752**	,796**	,641**	,894**
	Sig. (2-tailed)	,000	,001	,132	,007	,000	,004	,003	,001	,000	,508	,024	,026	,122	,000	,071		,018	,067	,001	,000	,025	,001	,000	,010	,000
	N	13	13	15	15	13	15	15	15	15	13	15	15	8	15	13	15	14	14	15	13	12	15	15	15	15
quercetin-3-glucoside	Pearson Correlation	,630*	,931**	,768**	,943**	,655*	-,722**	-,806**	-,853**	,891**	-,049	,901**	,887**	,805*	,932**	,943**	,619*	1	,969**	,929**	,910**	,542	,941**	,923**	,948**	,852**
	Sig. (2-tailed)	,021	,000	,001	,000	,011	,002	,000	,000	,000	,875	,000	,000	,029	,000	,000	,018		,000	,000	,000	,056	,000	,000	,000	,000
	N	13	14	15	15	14	15	15	15	15	13	15	15	7	15	14	14	15	15	15	14	13	15	15	15	15
kaempferol rhamnosylhexoside 1	Pearson Correlation	,523	,822**	,761**	,869**	,550*	-,537*	-,684**	-,713**	,775**	-,070	,800**	,783**	,820*	,831**	,944**	,502	,969**	1	,826**	,802**	,410	,836**	,804**	,854**	,723**
	Sig. (2-tailed)	,067	,000	,001	,000	,041	,039	,005	,003	,001	,820	,000	,001	,024	,000	,000	,067	,000		,000	,001	,164	,000	,000	,000	,002
	N	13	14	15	15	14	15	15	15	15	13	15	15	7	15	14	14	15	15	15	14	13	15	15	15	15
kaempferol-3-rutinoside	Pearson Correlation	,704**	,990**	,705**	,951**	,797**	-,890**	-,860**	-,949**	,958**	-,065	,932**	,919**	-,028	,979**	,856**	,769**	,929**	,826**	1	,978**	,655*	,979**	,976**	,956**	,936**
	Sig. (2-tailed)	,005	,000	,002	,000	,001	,000	,000	,000	,000	,825	,000	,000	,947	,000	,000	,001	,000	,000		,000	,015	,000	,000	,000	,000
	N	14	14	16	16	14	16	16	16	16	14	16	16	8	16	14	15	15	15	16	14	13	16	16	16	16
quercetin-3-glucuronide	Pearson Correlation	,768**	,985**	,689**	,928**	,813**	-,876**	-,799**	-,975**	,983**	-,214	,930**	,909**	-,377	,969**	,840**	,841**	,910**	,802**	,978**	1	,716**	,974**	,981**	,956**	,964**
	Sig. (2-tailed)	,002	,000	,006	,000	,001	,000	,001	,000	,000	,505	,000	,000	,461	,000	,000	,000	,000	,001	,000		,006	,000	,000	,000	,000
	N	13	14	14	14	13	14	14	14	14	12	14	14	6	14	13	13	14	14	14	14	13	14	14	14	14
kaempferol hexoside 1	Pearson Correlation	,524	,680*	,666*	,618*	,613*	-,652*	-,501	-,731**	,692**	-,243	,668*	,646*	-,387	,658*	,444	,639*	,542	,410	,655*	,716**	1	,656*	,663*	,640*	,667*
	Sig. (2-tailed)	,080	,011	,013	,025	,034	,016	,081	,005	,009	,471	,013	,017	,449	,015	,148	,025	,056	,164	,015	,006		,015	,013	,019	,013
	N	12	13	13	13	12	13	13	13	13	11	13	13	6	13	12	12	13	13	13	13	13	13	13	13	13
TOTAL	Pearson Correlation	,729**	,992**	,723**	,965**	,799**	-,884**	-,909**	-,944**	,964**	,033	,936**	,932**	,275	,983**	,859**	,752**	,941**	,836**	,979**	,974**	,656*	1	,991**	,977**	,948**
	Sig. (2-tailed)	,003	,000	,002	,000	,001	,000	,000	,000	,000	,912	,000	,000	,509	,000	,000	,001	,000	,000	,000	,000	,015		,000	,000	,000
	N	14	14	16	16	14	16	16	16	16	14	16	16	8	16	14	15	15	15	16	14	13	16	16	16	16
FRAP	Pearson Correlation	,793**	,997**	,679**	,945**	,816**	-,906**	-,906**	-,961**	,981**	-,012	,909**	,902**	-,505	,978**	,816**	,796**	,923**	,804**	,976**	,981**	,663*	,991**	1	,971**	,973**
	Sig. (2-tailed)	,001	,000	,004	,000	,000	,000	,000	,000	,000	,966	,000	,000	,202	,000	,000	,000	,000	,000	,000	,000	,013	,000		,000	,000
	N	14	14	16	16	14	16	16	16	16	14	16	16	8	16	14	15	15	15	16	14	13	16	16	16	16
ABTS	Pearson Correlation	,671**	,969**	,736**	,959**	,678**	-,869**	-,854**	-,946**	,922**	-,046	,932**	,918**	,542	,937**	,869**	,641**	,948**	,854**	,956**	,956**	,640*	,977**	,971**	1	,905**
	Sig. (2-tailed)	,009	,000	,001	,000	,008	,000	,000	,000	,000	,876	,000	,000	,165	,000	,000	,010	,000	,000	,000	,000	,019	,000	,000		,000
	N	14	14	16	16	14	16	16	16	16	14	16	16	8	16	14	15	15	15	16	14	13	16	16	16	16
DPPH	Pearson Correlation	,893**	,966**	,571*	,874**	,874**	-,881**	-,877**	-,937**	,990**	-,030	,828**	,823**	-,483	,946**	,742**	,894**	,852**	,723**	,936**	,964**	,667*	,948**	,973**	,905**	1
	Sig. (2-tailed)	,000	,000	,021	,000	,000	,000	,000	,000	,000	,920	,000	,000	,225	,000	,002	,000	,000	,002	,000	,000	,013	,000	,000	,000	
	N	14	14	16	16	14	16	16	16	16	14	16	16	8	16	14	15	15	15	16	14	13	16	16	16	16

*. Correlation is significant at the 0.05 level (2-tailed).

**. Correlation is significant at the 0.01 level (2-tailed).

c. Cannot be computed because at least one of the variables is constant.
