# Supplementary material for: Phytochemical Profile and Antioxidant Properties of Invasive Plants Ailanthus altissima (Mill.) Swingle and Helianthus tuberosus L. in Istria Region, Croatia
Source: Antioxidants (Basel). 2025 Jun 3;14(6):677. doi: 10.3390/antiox14060677 (PMC12189882; doi:10.3390/antiox14060677)
Supplement: Supplementary file 1 [file antioxidants-14-00677-s001.zip › SUPPLEMENTS/Table S5.pdf]

Table S5. Phenolic compounds (mg/g of dry weight (DW)) of *Ailanthus altissima* (Mill.) Swingle and *Helianthus tuberosus* L. leaf and flower extracts identified by LC-DAD-MS in two solvents, ethanol (EtOH) and methanol (MeOH). Values represent the mean ± SD of four replicates. Different letters (a, b, c, A, B, C) in the same row indicate significant intra-species differences, determined by Two-way ANOVA and Tukey's test, $p \leq 0.01$); nd = not detected.

Phenolic Compounds	*A. altissima*				*H. tuberosus*			
	LEAF 70% EtOH	LEAF 80% MeOH	FLOWER 70% EtOH	FLOWER 80% MeOH	LEAF 70% EtOH	LEAF 80% MeOH	FLOWER 70% EtOH	FLOWER 80% MeOH
3-caffeoylquinic acid 1	0.975 ± 0.275 [a]	0.540 ± 0.119 [a]	0.924 ± 0.190 [a]	0.934 ± 0.123 [a]	0.026 ± 0.002 [C]	0.013 ± 0.003 [C]	0.405 ± 0.094 [B]	0.644 ± 0.136 [A]
3-caffeoylquinic acid 2	2.917 ± 0.122 [b]	3.566 ± 0.258 [a]	n.d.	n.d.	n.d.	n.d.	n.d.	n.d.
4-caffeoylquinic acid 1	0.543 ± 0.119 [a]	0.471 ± 0.087 [a]	0.100 ± 0.017 [b]	0.157 ± 0.047 [b]	1.336 ± 0.183 [A]	1.573 ± 0.302 [A]	0.190 ± 0.064 [B]	0.272 ± 0.031 [B]
4-caffeoylquinic acid 2	3.112 ± 0.530 [a]	3.055 ± 0.144 [a]	0.449 ± 0.015 [b]	0.577 ± 0.142 [b]	n.d.	n.d.	n.d.	n.d.
5-caffeoylquinic acid 1	5.298 ± 0.474 [a]	6.512 ± 0.864 [a]	1.721 ± 0.421 [b]	2.852 ± 0.662 [b]	25.341 ± 1.831 [B]	33.926 ± 0.890 [A]	2.052 ± 0.261 [C]	3.341 ± 0.516 [C]
5-caffeoylquinic acid 2	9.080 ± 1.203 [a]	6.798 ± 0.638 [b]	n.d.	n.d.	0.673 ± 0.061 [A]	0.492 ± 0.224 [A]	0.097 ± 0.011 [B]	0.151 ± 0.035 [B]
caffeic acid	1.413 ± 0.314 [a]	1.422 ± 0.326 [a]	0.426 ± 0.029 [b]	0.220 ± 0.052 [b]	0.086 ± 0.014 [AB]	0.054 ± 0.001 [B]	0.100 ± 0.012 [AB]	0.157 ± 0.037 [A]
caffeic acid hexoside 1	0.374 ± 0.057 [a]	0.272 ± 0.021 [a]	0.089 ± 0.015 [b]	0.139 ± 0.042 [b]	0.439 ± 0.052 [AB]	0.299 ± 0.085 [B]	0.565 ± 0.109 [A]	0.492 ± 0.047 [AB]
caffeic acid hexoside 2	0.273 ± 0.086 [b]	0.152 ± 0.028 [b]	1.563 ± 0.100 [a]	2.110 ± 0.394 [a]	0.164 ± 0.024 [A]	0.122 ± 0.017 [A]	0.148 ± 0.024 [A]	0.167 ± 0.028 [A]
dicaffeoylquinic acid 1	n.d.	n.d.	n.d.	n.d.	0.551 ± 0.111 [A]	0.274 ± 0.021 [B]	0.205 ± 0.008 [B]	0.338 ± 0.043 [B]
dicaffeoylquinic acid 2	n.d.	n.d.	n.d.	n.d.	5.880 ± 0.584 [B]	9.042 ± 0.253 [A]	1.079 ± 0.043 [C]	1.778 ± 0.224 [C]
dicaffeoylquinic acid 3	n.d.	n.d.	n.d.	n.d.	0.599 ± 0.091 [AB]	0.323 ± 0.066 [C]	0.475 ± 0.019 [BC]	0.782 ± 0.099 [A]
dicaffeoylquinic acid 4	n.d.	n.d.	n.d.	n.d.	0.703 ± 0.076 [A]	0.788 ± 0.485 [A]	0.438 ± 0.077 [B]	0.750 ± 0.142 [A]
p-coumaric acid hexoside 1	0.461 ± 0.098 [a]	0.394 ± 0.003 [ab]	0.192 ± 0.033 [b]	0.300 ± 0.091 [ab]	n.d.	n.d.	n.d.	n.d.
3 p-coumaroylquinic acid	0.346 ± 0.090 [a]	0.294 ± 0.044 [a]	0.005 ± 0.001 [b]	0.031 ± 0.046 [b]	3.623 ± 0.485 [A]	1.032 ± 0.181 [B]	0.240 ± 0.013 [C]	0.153 ± 0.020 [C]
4-p-coumaroylquinic acid 1	0.788 ± 0.053 [ab]	1.239 ± 0.268 [a]	0.443 ± 0.038 [b]	0.794 ± 0.202 [ab]	0.153 ± 0.025 [A]	0.097 ± 0.018 [B]	0.078 ± 0.006 [B]	0.084 ± 0.015 [B]

4-p-coumaroylquinic acid 2	n.d.	n.d.	n.d.	n.d.	0.095 ± 0.014 A	0.090 ± 0.027 A	n.d.	n.d.
5-p-coumaroylquinic acid 1	0.530 ± 0.080 ab	0.559 ± 0.040 ab	0.424 ± 0.016 b	0.663 ± 0.072 a	0.485 ± 0.050 B	0.630 ± 0.107 A	n.d.	n.d.
5-p-coumaroylquinic acid 2	n.d.	n.d.	n.d.	n.d.	0.315 ± 0.043 A	0.344 ± 0.050 A	0.111 ± 0.025 B	0.051 ± 0.001 B
3-feruloylquinic acid	0.262 ± 0.067 b	0.273 ± 0.018 ab	0.399 ± 0.013 ab	0.512 ± 0.154 a	0.055 ± 0.008 C	0.060 ± 0.010 BC	0.115 ± 0.035 AB	0.150 ± 0.017 A
4-feruloylquinic acid	0.028 ± 0.002 b	0.039 ± 0.002 b	0.523 ± 0.026 a	0.510 ± 0.010 a	n.d.	n.d.	n.d.	n.d.
5-feruloylquinic acid 1	0.021 ± 0.003 c	0.025 ± 0.007 c	0.830 ± 0.032 b	1.297 ± 0.142 a	0.431 ± 0.036 AB	0.502 ± 0.103 A	0.242 ± 0.025 C	0.326 ± 0.055 BC
5-feruloylquinic acid 2	n.d.	n.d.	n.d.	n.d.	0.189 ± 0.024 A	0.198 ± 0.044 A	0.134 ± 0.038 A	0.174 ± 0.078 A
**Hydroxycinnamic acid derivatives**	**26.771 ± 1.684 a**	**25.610 ± 2.018 a**	**8.086 ± 0.444 b**	**10.761 ± 2.931b**	**41.131 ± 2.653 A**	**47.157 ± 7.205 A**	**6.615 ± 0.674 B**	**9.808 ± 1.300 B**
Gallic acid	0.531 ± 0.081 b	0.542 ± 0.057 b	0.947 ± 0.059 ab	1.300 ± 0.276 a	0.080 ± 0.193 A	0.121 ± 0.021 AB	n.d.	0.190 ± 0.051 A
Protocatechuic acid	0.026 ± 0.003 b	n.d.	n.d.	n.d.	1.503 ± 0.308 A	2.131 ± 0.433 A	0.404 ± 0.060 B	0.240 ± 0.086 B
Ellagic acid	22.486 ± 2.276 a	22.3683 ± 2.984 a	2.125 ± 0.397 b	2.207 ± 0.327 b	n.d.	n.d.	n.d.	n.d.
Ellagic acid pentoside 1	4.948 ± 0.494 a	4.597 ± 0.157 ab	3.074 ± 0.641 b	3.576 ± 0.413 ab	n.d.	n.d.	n.d.	n.d.
Ellagic acid pentoside 2	0.042 ± 0.008 b	0.051 ± 0.007 b	0.689 ± 0.063 a	0.615 ± 0.041 a	n.d.	n.d.	n.d.	n.d.
**Hydroxybenzoic acid derivatives**	**28.037 ± 2.767 a**	**25.991 ± 0.992 a**	**7.693 ± 0.881 b**	**6.976 ± 1.661 b**	**1.583 ± 0.292 A**	**2.192 ± 0.503 A**	**0.404 ± 0.060 B**	**0.430 ± 0.130 B**
procyanidin dimer 1	0.029 ± 0.007 b	0.024 ± 0.004 b	0.204 ± 0.050 a	0.338 ± 0.079 a	n.d.	n.d.	n.d.	n.d.
procyanidin dimer 2	4.373 ± 0.529 a	4.341 ± 0.173 a	n.d.	n.d.	n.d.	n.d.	n.d.	n.d.
Epicatechin	0.020 ± 0.0030 b	0.034 ± 0.007 b	5.661 ± 0.280 a	5.516 ± 1.076 a	n.d.	n.d.	n.d.	n.d.
Gallocatechin	2.331 ± 0.203 a	0.954 ± 0.092 b	0.790 ± 0.131 b	1.095 ± 0.214 b	n.d.	n.d.	n.d.	n.d.
**Flavanols**	**6.779 ± 0.526 ab**	**5.393 ± 0.196 b**	**6.838 ± 0.271 ab**	**7.192 ± 1.067 a**	**n.d.**	**n.d.**	**n.d.**	**n.d.**
Quercetin pentoside 1	0.336 ± 0.035 ab	0.099 ± 0.028 c	0.286 ± 0.040 bc	0.160 ± 0.019 a	n.d.	n.d.	0.132 ± 0.027 A	0.154 ± 0.070 A

Quercetin pentoside 2	0.011 ± 0.002 ^{b}	0.014 ± 0.001 ^{b}	0.032 ± 0.007 ^{a}	0.030 ± 0.005 ^{a}	n.d.	n.d.	0.138 ± 0.011 ^{A}	0.072 ± 0.012 ^{B}
Quercetin-3-rutinoside	0.001 ± 0.000 ^{a}	0.001 ± 0.000 ^{a}	n.d.	n.d.	1.279 ± 0.196 ^{A}	1.279 ± 0.105 ^{A}	0.262 ± 0.075 ^{B}	0.342 ± 0.053 ^{B}
Quercetin-3-galactoside	1.926 ± 0.195 ^{a}	1.929 ± 0.210 ^{a}	2.634 ± 0.366 ^{a}	2.164 ± 0.125 ^{a}	0.489 ± 0.085 ^{A}	0.245 ± 0.022 ^{B}	0.124 ± 0.019 ^{B}	0.116 ± 0.021 ^{B}
Quercetin-3-glucoside	8.031 ± 0.813 ^{a}	7.678 ± 0.596 ^{a}	2.940 ± 0.550 ^{b}	3.054 ± 0.452 ^{b}	0.760 ± 0.073 ^{A}	0.871 ± 0.083 ^{A}	0.422 ± 0.050 ^{B}	0.425 ± 0.118 ^{B}
Quercetin-3-glucuronide	n.d.	n.d.	n.d.	n.d.	5.011 ± 0.227 ^{A}	4.736 ± 0.291 ^{A}	1.634 ± 0.140 ^{B}	0.850 ± 0.143 ^{C}
Quercetin-3-rhamnoside	0.140 ± 0.026 ^{b}	0.158 ± 0.016 ^{b}	1.004 ± 0.075 ^{a}	1.028 ± 0.094 ^{a}	n.d.	n.d.	n.d.	n.d.
Quercetin galloylhexoside 1	0.283 ± 0.032 ^{b}	0.250 0.038 ^{b}	0.617 ± 0.053 ^{a}	0.237 0.082 ^{b}	n.d.	n.d.	n.d.	n.d.
Quercetin galloylhexoside 2	0.929 ± 0.179 ^{a}	1.119 ± 0.522 ^{a}	0.953 ± 0.087 ^{a}	0.852 ± 0.057 ^{a}	n.d.	n.d.	n.d.	n.d.
Quercetin acetylhexoside 1	3.469 ± 0.346 ^{a}	3.526 ± 0.306 ^{a}	1.010 ± 0.109 ^{b}	1.209 ± 0.111 ^{b}	n.d.	n.d.	n.d.	n.d.
Quercetin acetylhexoside 2	0.175 ± 0.023 ^{b}	0.227 ± 0.025 ^{b}	0.363 ± 0.030 ^{a}	0.161 ± 0.026 ^{b}	n.d.	n.d.	n.d.	n.d.
Quercetin-rhamnosylhexoside	n.d.	n.d.	n.d.	n.d.	0.235 ± 0.006 ^{A}	0.290 ± 0.023 ^{A}	n.d.	n.d.
Quercetin malosyl hexoside	n.d.	n.d.	n.d.	n.d.	n.d.	n.d.	0.558 ± 0.004 ^{B}	0.801 ± 0.106 ^{A}
Isorhamnetin hexoside	n.d.	n.d.	n.d.	n.d.	0.240 ± 0.016 ^{A}	0.269 ± 0.026 ^{A}	0.200 ± 0.032 ^{A}	0.200 ± 0.045 ^{A}
Isoramnetin hexosylpentoside	n.d.	n.d.	n.d.	n.d.	0.470 ± 0.035 ^{AB}	0.548 ± 0.071 ^{A}	0.351 ± 0.063 ^{B}	0.338 ± 0.066 ^{B}
Isorhamnetin glucuronide	n.d.	n.d.	n.d.	n.d.	0.374 ± 0.025 ^{A}	0.419 ± 0.041 ^{A}	0.184 ± 0.043 ^{B}	0.197 ± 0.030 ^{B}
Isorhamnetin acetylhexoside	0.214 ± 0.059 ^{b}		0.555 ± 0.113 ^{a}		n.d.	n.d.	n.d.	n.d.
Kaempferol hexoside 1	1.190 ± 0.120 ^{b}	1.191 ± 0.130 ^{b}	2.075 ± 0.288 ^{a}	1.704 ± 0.099 ^{a}	0.141 ± 0.007 ^{A}	0.133 ± 0.008 ^{A}	0.117 ± 0.026 ^{A}	0.096 ± 0.014 ^{A}
Kaempferol hexoside 2	0.751 ± 0.119 ^{a}	0.871 ± 0.075 ^{a}.	n.d.	0.148 ± 0.041 ^{b}.	n.d.	1.429 ± 0.053 ^{B}	0.055 ± 0.002 ^{C}	0.091 ± 0.011 ^{C}
Kaempferol rhamnosylhexoside 1	0.087 ± 0.017 ^{c}	0.083 ± 0.010 ^{c}	0.282 ± 0.034 ^{b}	0.521 ± 0.068 ^{a}	0.048 ± 0.005 ^{AB}	0.056 ± 0.005 ^{A}	0.036 ± 0.0047 ^{B}	0.036 ± 0.010 ^{B}
Kaempferol rhamnosylhexoside 2	0.123 ± 0.021 ^{c}	0.164 ± 0.019 ^{bc}	0.304 ± 0.069 ^{ab}	0.322 ± 0.060 ^{a}	n.d.	n.d.	n.d.	n.d.

Kaempferol-3-rutinoside	n.d.	n.d.	n.d.	n.d.	0.918 ± 0.155 A	0.984 ± 0.068 A	0.211 ± 0.072 B	0.149 ± 0.019 B
Kaempferol-3-glucuronide	n.d.	n.d.	n.d.	n.d.	7.125 ± 0.639 A	7.155 ± 0.236 A	0.420 ± 0.054 B	0.201 ± 0.054 B
Kaempferol acetylhexoside 1	n.d.	0.210 ± 0.031 b	n.d.	0.339 ± 0.014 b	0.474 ± 0.078 A	0.312 ± 0.112 AB	0.132 ± 0.026 B	0.149 ± 0.062 B
Kaempferol acetylhexoside 2	0.736 ± 0.072 a	0.714 ± 0.063 a	0.594 ± 0.073 a	0.601 ± 0.134 a	n.d.	n.d.	n.d.	n.d.
Kaempferol galloylhexoside	0.202 ± 0.035 ab	0.256 ± 0.033 a	0.134 ± 0.029 b	0.128 ± 0.022 b	n.d.	n.d.	n.d.	n.d.
**Flavonols**	**18.521 ± 1.489 a**	**16.223 ± 3.192 a**	**14.119 ± 1.376 a**	**12.655 ± 1.219 a**	**23.132 ± 1.197 A**	**18.734 ± 0.742 C**	**4.973 ± 0.244 C**	**4.214 ± 0.678 B**
Naringenin hexoside 1	0.366 ± 0.048 ab	0.334 ± 0.026 b	0.656 ± 0.077 ab	0.607 ± 0.175 a	n.d.	n.d.	n.d.	n.d.
Naringenin hexoside 2	0.033 ± 0.007 a	0.032 ± 0.004 ab	0.019 ± 0.002 b	0.035 ± 0.005 a	n.d.	n.d.	n.d.	n.d.
Naringenin hexoside 3	0.046 ± 0.008 a	0.052 ± 0.006 a	0.023 ± 0.002 b	0.023 ± 0.002 b	n.d.	n.d.	n.d.	n.d.
Naringenin hexoside 4	0.332 ± 0.053 b	0.302 ± 0.048 b	0.572 ± 0.033 a	0.646 ± 0.033 a	n.d.	n.d.	n.d.	n.d.
**Flavanones**	**0.776 ± 0.086 ab**	**0.635 ± 0.125 b**	**1.269 ± 0.086 a**	**1.159 ± 0.340 ab**	**n.d.**	**n.d.**	**n.d.**	**n.d.**
Vescalagin isomer 1	80.705 ± 4.531 b	123.309 ± 21.401 a	54.298 ± 6.480 b	82.107 ± 8.290 b	n.d.	n.d.	n.d.	n.d.
Vescalagin isomer 2	13.442 ± 0.706 b	18.538 ± 0.043 b	27.364 ± 1.128 a	31.937 ± 3.180 a	n.d.	n.d.	n.d.	n.d.
HHDP galloylhexose	11.989 ± 1.830 a	11.675 ± 1.140 a	8.659 ± 0.500 ab	7.821 ± 0.845 b	n.d.	n.d.	n.d.	n.d.
HHDP digalloylhexose isomer	1.749 ± 0.3436 b	0.627 ± 0.006 a	0.715 ± 0.029 c	0.834 ± 0.083 c	n.d.	n.d.	n.d.	n.d.
**Ellagitannins**	**107.431 ± 4.019 b**	**142.648 ± 16.676 a**	**91.035 ± 6.712 b**	**122.698 ± 8.490 ab**	**n.d.**	**n.d.**	**n.d.**	**n.d.**
Apigenin hexoside	0.669 ± 0.107 b	0.845 ± 0.029 a	0.142 ± 0.010 c	0.152 ± 0.002 c	n.d.	n.d.	n.d.	n.d.
**Flavones**	**0.669 ± 0.107 b**	**0.845 ± 0.029 a**	**0.142 ± 0.010 c**	**0.152 ± 0.002 c**	**n.d.**	**n.d.**	**n.d.**	**n.d.**
**TOTAL**	**189.541 ± 9.4473 b**	**234.747± 26.9073 a**	**129.182 ± 7.002 c**	**163.128 ± 11.371 bc**	**65.841 ± 2.560 A**	**70.835 ± 4.843 A**	**12.048 ± 0.741 B**	**14.451 ± 1.855 B**

The external standards used: caffeic acid, apigenin-7-glucoside, ferulic acid, quercetin-3-O-rhamnoside, neochlorogenic (3-caffeoylquinic) acid, naringenin, ellagic acid, gallic acid, chlorogenic acid, and rutin (quercetin-3-O-rutinoside); (-)epicatechin, quercetin-3-O-galactoside, quercetin-3-O-glucoside, p-coumaric acid, procyanidin B1, and kaempferol-O-glucoside; quercetin-3-O-xyloside and quercetin-3-O-arabinopyranoside; and isorhamnetin-3- O-glucoside.
